# Diabetes Mellitus and Cardiopulmonary Bypass (CPB): Pathophysiological Mechanisms Related to Inflammation and Cardiovascular Disease

**DOI:** 10.3390/cimb47110911

**Published:** 2025-11-02

**Authors:** Theodora M. Stougiannou, Theocharis Koufakis, Nikolaos Papanas, Dimos Karangelis

**Affiliations:** 1 Department of Cardiothoracic Surgery, Democritus University of Thrace University General Hospital, Democritus University of Thrace, 68132 Alexandroupolis, Greece; theodorastougiannos@gmail.com; 2 Second Propedeutic Department of Internal Medicine, Hippokration General Hospital, Aristotle University of Thessaloniki, 54642 Thessaloniki, Greece; thkoyfak@auth.gr; 3 Diabetes Centre, Second Department of Internal Medicine, Democritus University of Thrace University General Hospital, Democritus University of Thrace, 68132 Alexandroupolis, Greece

**Keywords:** diabetes mellitus, inflammation, cardiac, surgery, cardiopulmonary bypass

## Abstract

Type 2 Diabetes Mellitus (T2DM) is a chronic disease caused by the resistance of tissues to the actions of insulin as well as the progressive failure to produce adequate amounts of insulin in pancreatic β-cells. Research has further shown that T2DM is characterized by a generalized state of low-grade inflammation; this inflammation is often related to overnutrition and obesity leading to an excess storage of lipid particles in adipose cells. Eventually, this will stimulate the pathophysiological pathways of cellular stress and inflammation. The inflammation characterizing T2DM can then contribute, along with other mechanisms of hyperglycemia, to the emergence of cardiovascular disease. Due to the resulting heart disease, many patients with T2DM may be inevitably required to undergo cardiac surgery with cardiopulmonary bypass (CPB), a process also characterized by an intense inflammatory response with possible effects and disruptions in immune system functions. It is thus the purpose of this narrative review to summarize and present evidence in the literature related to the inflammatory interplay occurring between T2DM, cardiovascular disease, and cardiac surgery with CPB.

## 1. Introduction

Diabetes mellitus (DM) is a chronic disease related to scarcity/absence or the reduced effectiveness of insulin throughout target tissues; it is estimated to affect ~14% of all adults older than 18 years, according to 2022 statistics from the World Health Organization (WHO) evaluating global disease burden [1]. DM can affect multiple organ systems, including the heart and blood vessels. In particular, cardiovascular disease affects a relatively large subset of those affected by DM, estimated at ~46% in North America and the Caribbean and at ~42.5% in South East Asia [2]. Many pathophysiological associations can be made between cardiovascular disease and Type 2 Diabetes Mellitus (T2DM), in particular, some of which can be attributed to the generalized low-grade inflammation characterizing T2DM and the resulting inflammatory sequelae across systems [3]. Patients with T2DM may be subjected to cardiac surgery procedures and as such are exposed to cardiopulmonary bypass (CPB). Both the trauma of the surgery itself as well as the use of CPB can elicit significant inflammation postoperatively [4] or stimulate postoperative hyperglycemia and insulin consumption. The latter effect is often augmented in T2DM [5].

The purpose of this narrative review is to describe and summarize the inflammatory and immune mechanisms contributing to T2DM and associated cardiovascular disease. A review of the relevant literature has been carried out, spanning 1990–2025, across PubMed and Google Scholar. Keywords used include (diabetes OR type 2 diabetes OR diabetes mellitus OR diabetic) AND (inflammation OR inflammatory) AND (cardiac OR cardiovascular OR heart), (inflammation OR inflammatory) AND (cardiopulmonary bypass) AND (cardiac surgery), (diabetes OR type 2 diabetes OR diabetes mellitus OR diabetic) AND (cardiopulmonary bypass) AND (cardiac surgery), (cardiopulmonary bypass) AND (inflammation OR inflammatory). We apply a wide search scope, ranging from 1990 up to 2025, as noted previously; this strategy aims to identify when study into a particular pathophysiologic phenomenon begins, and how, with additional similar research throughout the years, more information to explain the initial problem is gathered. This allows for a more spherical understanding of biological and physiological research relevant to cardiopulmonary bypass and cardiac surgery.

In general, this text thus aims to evaluate the pathophysiological relationship between inflammation and the general inflammatory status characterizing DM, including T2DM, how these inflammatory mechanisms contribute to the cardiovascular manifestations of DM, and finally, how these may interact with the effects on the immune system instigated by CPB during cardiac surgery.

## 2. Diabetes Mellitus: Classification, Risk Factors, and Cardiovascular Associations

Diabetes mellitus (DM) comprises a group of disorders that affect glucose metabolism; in these, glucose can be underutilized or overproduced, eventually leading to hyperglycemia. Disease categories thus include type 1 diabetes mellitus (T1DM), T2DM, gestational diabetes mellitus (GDM), and diabetes mellitus due to pancreatic disorders (type 3c diabetes mellitus [T3DM]) or medications, as well as DM due to genetic mutations [6]. T2DM, in particular, is caused by the resistance of tissues to insulin and the progressive functional failure of pancreatic β-cells. Dysregulation and abnormalities in lipid metabolism/trafficking (dyslipidemia) can occur as well. Patients often exhibit increased levels of small and dense low-density lipoprotein (LDL)/cholesterol particles, non-high density lipoprotein (HDL)/cholesterol particles, as well as lipoproteins containing apolipoprotein B and triglycerides [7]. This observed dyslipidemia, a risk factor for T2DM, constitutes a risk factor for obesity as well [8]. Furthermore, non-alcoholic fatty liver disease (NAFLD), defined as the excessive accumulation of lipids in the liver, in association with metabolic syndrome, is in turn associated with an increased risk of both cardiovascular disease and T2DM. Metabolic syndrome can be generally defined as a group of metabolic abnormalities which includes dyslipidemia, central obesity, insulin resistance, and hypertension [9]. Finally, both T2DM as well as chronic kidney disease, defined as persistent kidney injury or an estimated glomerular filtration rate (eGFR) < 60 mL/min/1.73 m^2^ for more than 3 months, are independent risk factors for the development of adverse cardiovascular events in patients with known cardiovascular disease [10].

Though T2DM has been classically associated with cardiovascular complications [11], other forms of DM can be associated with cardiovascular sequelae as well. In T1DM, for example, which usually manifests earlier than T2DM and is driven by different underlying mechanisms (autoimmune destruction of pancreatic β-cells) [12], cardiovascular disease can also occur. Cardiovascular morbidity and mortality in T1DM increases if the age of onset is <10 years of age; usual risk factors for cardiovascular disease also contribute to risk here as well, including tobacco, LDL-cholesterol, and hypertension [13]. For example, studies evaluating T1DM with a median follow-up of 10 years report a cardiovascular risk increase of 3.85 for patients that develop T1DM at ages 26–30; conversely, a risk increase of 11.44 is reported for patients that develop the condition before 10 years of age [14]. Contrary to T2DM, however, cardiovascular disease risk in T1DM remains high even with adequate control of glucose levels, hinting at other etiological factors and different underlying mechanisms, including hypoglycemia, variability in glucose levels, as well as autoimmunity [13]. Additional forms of DM and their associations with cardiovascular disease are included in Table 1 (Table 1).

## 3. Metabolism and Inflammation: The Inflammatory State Characterizing Type 2 Diabetes Mellitus with Cardiovascular Pathophysiology Associations

### 3.1. Metabolism and Inflammation: The Generalized Inflammatory State of Type 2 Diabetes Mellitus with a Focus on Macrophages and T-Lymphocyte Populations

Glucose homeostasis/availability is mainly regulated by adipose tissue, skeletal muscle, and liver; glucose metabolism and the effects of insulin within these sites determines the levels of insulin resistance. Within adipose tissue and skeletal muscle, insulin resistance manifests as increased lipolysis and glucose intolerance, while in the liver, it results in high levels of fasting serum glucose [31]. Insulin signaling is carried out via the insulin receptor, which associates with and phosphorylates the insulin receptor substrate-1 (IRS-1). This stimulates phosphoinositide-3-kinase (PI3K)/protein kinase B (AKT) signaling pathways [32]. As a result, PI3K will induce the translocation of glucose transporter 4 (GLUT4) to the cell membrane surface and facilitate glucose uptake [33], while AKT will phosphorylate the forkhead box O1 (FOXO1) transcription factor. Forkhead box (FOX) transcription factors are expressed in various tissues and carry out diverse functions, with roles identified in cellular proliferation/apoptosis and inflammation. More specifically, in metabolism, FOXO1 regulates the expression of enzymes involved in gluconeogenesis, including phosphoenolpyruvate carboxykinase (PEPCK) and glucose-6-phosphatase (G6Pase). FOXO1 phosphorylation induced by insulin removes the FOX factors from the nucleus, thus inhibiting gluconeogenesis [34]. AKT can then phosphorylate glycogen synthase kinase 3 (GSK3), thereby directly stimulating glycogen synthesis [35]. As a result, suppression on mammalian target of rapamycin (mTOR) is removed, stimulating anabolic protein pathways [36].

Development of insulin resistance and pancreatic islet β-cell dysfunction are two of the main hallmarks of T2DM. Early in the natural history of the disease, mechanisms include impairment in insulin synthesis/secretion from pancreatic β-islet cells. As the disease progresses, glucose and lipid compounds accumulate, impairing survival of the β-islet cells themselves [37]. Thus, while early on, increased glucose levels stimulate continuous insulin production, secretion is eventually impaired due to physical consumption and effects on the levels of relevant transcription factors. Several factors have been shown to contribute to the emergence of insulin resistance, including genetic [38], epigenetic factors [39], as well as obesity [40]. Obesity confers an increased risk for a plethora of health conditions, including hypertension, hyperlipidemia, cardiovascular disease, and T2DM [41]. Obesity-associated inflammation has both innate and adaptive immunity components and represents a mechanistic link between obesity and T2DM [42]. Inflammatory factors and cytokines will be released both in adipose tissue as well as systemically, disrupting insulin signaling in multiple systems, eventually contributing to the systemic effects of T2DM [43].

Adipose tissue inflammation, owing to the adipose tissue hypertrophy [44] generated by an excess of triacylglycerol (TAG) storage will eventually contribute to adipose macrophage polarization towards the M1 phenotype. This promotes the establishment of an inflammatory microenvironment via the secretion of signaling factors including the interleukins (IL) IL-1β and IL-6, tumor necrosis factor alpha (TNF-α), monocyte chemoattractant protein-1 (MCP-1), and plasminogen activator inhibitor-1 (PAI-1) [45,46]. Activation of the chaperone protein CHOP due to endoplasmic reticulum (ER) stress also downregulates the secretion of IL-13, peroxisome proliferator activated receptor gamma protein (PPARγ), and adiponectin, as well as the secretion of Th2-lymphocyte cytokines (IL-4, IL-13), which would normally function to prevent excessive inflammatory system activation. There is also reduced polarization towards the M2 macrophage phenotype, while IL-13-mediated adipocyte differentiation is prevented as well [45]. Furthermore, due to a systems-wide shift towards pro-inflammatory M1-macrophage phenotypes, as well as local metabolite and reactive oxygen species (ROS) accumulation triggering IL-1β secretion [47], pro-inflammatory macrophages are often seen invading the pancreatic islets. Eventually, this contributes to the overall pancreatic islet inflammation and the clinical manifestations of T2DM [48]. Obesity and the resulting adipose tissue inflammation thus eventually results in a generalized, low-grade, systemic inflammation with the elevation of pro-inflammatory cytokines (C-reactive protein [CRP], TNF-α) [49]. The burden of overall adipose tissue inflammation, including levels of macrophages and IL-6, correlates with the levels of circulating CRP and TNF-α [50].

Increases in CD4+ T-lymphocyte populations have been detected within adipose tissues in animal models of T2DM, expressing high levels of CD44 molecule (CD44) along with programmed cell death protein-1 (PD-1) and CD153. These T-lymphocytes also exhibit characteristics of cellular senescence [51]. Similarly to the pro- and anti-inflammatory polarization paradigms previously described, Th1-lymphocytes are upregulated in the adipose tissue of T2DM patients [52]. They secrete interferon gamma (IFN-γ), which further contributes to the upregulation of other pro-inflammatory genes in adipose tissues, including TNF-α and MCP-1. These augment the accumulation of inflammatory cells even further, affecting glucose tolerance as well [53]. In general, within the adipose tissues of animals fed a high-fat diet, pro-inflammatory lymphocyte groups such as Th1-, γδT-, and CD8+ T-lymphocytes are relatively increased in relation to groups that function to resolve or attenuate immune responses, such as natural killer (NK) cells, Th2-, and Treg-lymphocytes [54] (Table 2).

### 3.2. Metabolism and Inflammation: The Generalized Inflammatory State of Type 2 Diabetes Mellitus with Cardiovascular Pathophysiology Associations

Macrophages, including tissue-resident macrophages, play key roles in the perpetuation of the generalized inflammation associated with T2DM [61]; though this can have widespread results throughout body systems, the effects of macrophages in cardiovascular systems will be examined in this narrative review. Under conditions of hyperglycemia, macrophages derived from the bone marrow exhibit higher levels of baseline IL-1β expression compared to populations under physiologic conditions. Upon stimulation by endotoxin (lipopolysaccharide [LPS]) and IFN-γ, they exhibit higher levels of TNF-α expression as well, along with reduced nitric oxide (NO) production [62]. In addition, when stimulated with IL-4 under hyperglycemic conditions, they will also express higher levels of arginase-1 (Arg-1) and IL-10, though their phagocytic and bactericidal activity is usually impaired [62]. Thus, in general, though macrophages in T2DM are more sensitized to produce pro-inflammatory cytokines, an event that contributes to the development of diabetic complications and inflammatory injury, their ability to clear infections is diminished [62]. Macrophages also undergo metabolic reprogramming under conditions of T2DM. This is a microenvironment characterized by high levels of pro-inflammatory cytokines (IL-1β, IL-6, and IFNγ), which then triggers the increased expression of pyruvate kinase M2 (PKM2). A metabolic switch towards anaerobic glycolysis thus occurs. At the same time, there is the reduced expression of citric-acid cycle (tricarboxylic acid cycle [TCA]) enzymes and downregulation of oxidative phosphorylation. This contributes to the generation of advanced glycation end-products (AGE) and ROS. Finally, there is also upregulation of hypoxia inducible factor-1 subunit alpha (HIF-1α) [63].

In the case of vascular disease, AGE/receptor for advanced glycation end-products (RAGE) activation on the surface of monocytes/macrophages contributes to the immune activation and production of ROS. AGE formation is accelerated by hyperglycemia, leading to their accumulation around blood vessels [64]. Activation of AGE/RAGE further stimulates intracellular signaling cascades that induce transforming growth factor beta (TGF-β), nuclear factor-Kappa B subunit 1 (NF-κB), mitogen-associated protein kinase (MAPK), and nicotinamide adenine dinucleotide phosphate (NADPH) oxidases (NOX), triggering the production of pro-inflammatory cytokines and vascular endothelial growth factor (VEGF), as well as the upregulation of adhesion molecules on the endothelial cell surface [65]. These adhesion molecules will then enhance entry of inflammatory cells into the vascular wall, contributing to vascular inflammation [66]. Macrophages are polarized towards M1 phenotypes, propagating this inflammation and contributing to atherosclerotic plaque instability [58]. LDL, present in these atherosclerotic plaques, can be modified not only by oxidation, as it often occurs in atherosclerosis, but by glycation as well, enhancing the interaction with subendothelial proteoglycan. In turn, this enhances phagocytosis and the release of pro-inflammatory cytokines (TNF-α, IL-1β, and IL-6) along with matrix metalloproteinase (MMP) [67]. Adiponectin normally functions to influence the macrophage polarization spectrum towards M2 phenotypes via adiponectin receptor-1 (AdipoR1) activation; this too, is reduced due to the ongoing adipose tissue inflammation. This will then translate to the augmentation of M1 macrophage polarization in the affected vascular wall as well [68].

AGE/RAGE activation also occurs on the surface of T2DM cardiomyocytes; this results in the activation of pro-inflammatory pathways via NF-κB signaling [69]. Due to impaired insulin receptor function, there is greater availability of the fatty acid translocase (CD36) receptor compared to GLUT4. This eventually leads to the accumulation of fatty acids within cardiomyocytes due to impairment of glycolysis combined with excessive fatty acid oxidation. As a result, toxic lipid intermediaries also accumulate [70]. The resulting ER stress eventually leads to cardiomyocyte death and release of danger-associated molecular patterns (DAMP) in the local microenvironment. This, in turn, leads to increased chemotaxis of pro-inflammatory monocytes and increased M1 macrophage polarization, further triggering pro-inflammatory pathways [71]. Fibrosis will also be stimulated, in part via the secretion of IL-1β by pro-inflammatory macrophages, leading to cardiac fibroblast activation [71].

T-lymphocyte alterations occur in T2DM as well, imbalances which could reflect the higher propensity for inflammation observed in T2DM [72]. Some studies additionally report a general reduction in naïve CD4+ T-lymphocytes, possibly corresponding to the chronic low-grade immune system activation in T2DM [73]. More specifically, Th1-lymphocyte activity is upregulated in the peripheral blood of T2DM patients as well as patients with prediabetes, though Th1-lymphocyte activity does not correlate directly with higher activity of Th17- and Th22-lymphocyte populations [74]. In addition, higher levels of memory T-lymphocytes, along with the lower levels of naïve T-lymphocytes observed during this time, generally point to chronic adaptive immune system activation and exhaustion [74]. In the case of diabetic cardiomyopathy, Th1-lymphocytes, via integrin-α4 interactions, will also activate cardiac fibroblasts, contributing to fibrosis [75]. On the other hand, administration of Treg-lymphocytes in animal models of diabetic cardiomyopathy has been shown to ameliorate cardiac fibrosis and cardiomyocyte hypertrophy via reductions in the levels of inflammation, macrophage accumulation, and cardiomyocyte apoptosis [76]. These effects point to a contribution of Treg-lymphocytes in diabetic cardiomyopathy, possibly due to absence or reduced function, as T2DM has been generally associated with reduced levels of Treg-lymphocytes [76] (Table 3).

## 4. Cardiopulmonary Bypass (CPB) and Inflammation: General Overview of Cardiopulmonary Bypass with a Focus on Macrophage, T-Lymphocyte Populations

### 4.1. Cardiopulmonary Bypass: Overview of the Circuit, Effects of Cardioplegic Arrest on Tissue Structure and Function

Cardiopulmonary bypass (CPB) is often used as a tool to operate on thoracic viscera, as it allows for the replacement of heart and lung function for a relatively short period of time. The circuit is composed of arterial and venous cannulation systems, a reservoir which functions as a chamber for venous return and a cardiotomy reservoir for aspirated blood originating in the surgical field. In addition, there are systems aimed at oxygenating and increasing the temperature of the circulating blood, pumps to facilitate flow within the overall system, filters to prevent circulation of emboli, vents to aid cardiac decompression, and finally, a system to deliver cardioplegic solutions. The latter is delivered via a separate system with its own reservoir, heat exchanger, pump, and filter [88].

Cardioplegia can be generally described as a technique used to induce cardiac arrest in the operated myocardium. A blood-free surgical field is additionally generated via cross-clamping of the aorta for a specified amount of time during CPB [88]. Aorta cross-clamping is associated with ischemic injury to the myocardium; this usually leads to the accumulation of by-products such as lactate, causing metabolic acidosis [89,90]. At the same time, calcium (Ca^2+^) handling is impaired, affecting cardiomyocyte contraction, while the activation of signaling pathways causing caspase-3 activation will stimulate cardiomyocyte apoptosis pathways as well [91]. The reintroduction of perfusion after cross-clamp removal will further accentuate tissue insult and lead to the production/accumulation of ROS, intracellular Ca^2+^ increase, stimulation of inflammatory signaling pathways involving both neutrophil and macrophage responses, and finally, stimulation of cardiomyocyte cell death pathways [91]. Aorta cross-clamp can also affect the biomechanical properties of the aortic wall itself, though no histological changes have been observed in associated studies [92]. However, some studies do show an association of aorta cross-clamping with pressure-induced aortic injury and late aortic sequelae, such as aortic rupture [93]. In essence, cardioplegic arrest and aorta cross-clamping recapitulate ischemia/reperfusion (I/R) tissue injury.

Cardioplegic solutions used during the procedure, are a method of pharmacological myocardial protection aimed at introducing temporary cardiac arrest and alleviating some of the associated/predicted tissue injury [94]. They can have varying compositions, with blood cardioplegia generally comprising a mixture of blood and crystalloid solution and crystalloid cardioplegic solutions with no blood admixture [95]. Many of these solutions often possess potassium (K^+^) concentrations of about 10–30 mmol/L and can cause hyperkalemia when introduced onto living myocardial tissue, depolarizing the cardiomyocyte cellular membranes to a new, less negative membrane potential. This induces depolarizing cardiomyocyte arrest and prevents voltage-dependent sodium (Na^+^) channel activation. Thus, with extracellular-type cardioplegic solutions, the heart is arrested in diastole [96]. On the other hand, non-depolarizing solutions containing adenosine, lidocaine, and magnesium (Mg^2+^) can be used as well [97]. Both aorta cross-clamp and aorta cross-clamp I/R combined with cardioplegia and CPB can lead to I/R injury and cardiomyocyte apoptosis, although signaling pathways differ [98]. Cardioplegic solutions can prevent some of this I/R injury via effects on protein kinase C (PKC) enzymes; more specifically, cardioplegic arrest is associated with an increase in PKC enzyme activity, including PKCδ and PKCε, and localization of these enzymes to the cardiomyocyte z-line as well the cell membrane surface. However, the latter event has been described as biologically insignificant [99]. A protective effect has been attributed to the upregulation of PKCε activity, an enzyme stimulated by hypoxia that can also prevent apoptosis due to I/R [99]. The latter occurs via inhibition of the pro-apoptotic proteins Bad and Bax, as well as inhibition of the mitochondrial permeability transition pore (MPTP) opening [99].

As already stated, cardioplegic arrest is associated with ischemic and I/R injury and can induce cardiomyocyte apoptosis via signaling pathways involving ischemia/hypoxia, the upregulation of ROS, and inflammation [100]. Concurrent application of lower temperatures with cardioplegia aids cardiomyocyte survival and prevents some of the cardioplegia-induced cardiomyocyte apoptosis [101]. This is ensured via the phosphorylation of AKT and the subsequent phosphorylation and inactivation of downstream enzyme targets such as GSK3β and Bad, leading to the downregulation of β-catenin and Bcl-2 [102]. Hypothermia also downregulates caspase-3 activity [102]. Furthermore, cardioplegic arrest induces mitochondrial stress, which is also involved in the stimulation of cardiomyocyte apoptosis via the activation of Bax and mitochondrial permeabilization [103]. Rates of cardiomyocyte apoptosis have been shown to be higher with cold blood and cold crystalloid cardioplegic solutions as opposed to tepid blood cardioplegia [104]. A prominent aspect of this myocardial I/R injury during cardioplegic arrest is mitochondrial dysfunction. During the ischemic phase, the MPTP is primed, although still closed; with reperfusion, ROS are produced and the pore opens, disrupting the mitochondrial membrane electrochemical gradient as well as any associated membrane proteins that contribute to respiration and adenosine triphosphate (ATP) synthesis [105,106]. Degree of injury and cardiomyocyte apoptosis depends on the number of affected mitochondria; localized MPTP disturbances (10–50% of total mitochondria) may lead to either recovery or cardiomyocyte apoptosis, whilst widespread effects (about 50–90% of mitochondria) often lead to cardiomyocyte necrosis [106]. Cardiac tissue inflammation is observed as well, characterized by an increase in the pro-inflammatory cytokines TNF-α, IL-6, and IL-8, identified after use of both cold blood and cold crystalloid cardioplegia solutions [103].

Cardioplegic solutions used for myocardial protection may also have detrimental effects themselves, especially with multiple infusions. Risk for occurrence of I/R injury exists during each infusion, as well as after the aortic cross-clamp is removed [107]. Solutions with a high K^+^ concentration can lead to Ca^2+^ overload, even if they do not contain any Ca^2+^ components; this so-called Ca^2+^ paradox is generally described as severe myocardial tissue damage following the reintroduction of normal Ca^2+^ levels in a cardiomyocyte, which resumes function [108]. Cardioplegia solutions can also lead to endothelial injury, mostly due to the failure of appropriate vascular smooth muscle cell (VSMC) and endothelial cell protection, often contributing to endothelial dysfunction, which is then followed by VSMC dysfunction [109]. Endothelial dysfunction with cardioplegic solutions has also been attributed to neutrophils chemotaxis and the stimulation of inflammatory pathways [110]. Use of cardioplegic solutions containing histidine, tryptophan, and ketoglutarate (HTK) or blood cardioplegic solutions with HTK have been shown to better preserve coronary endothelial function and the endothelium-dependent relaxation response to acetylcholine. On the other hand, solutions such as Del Nido cardioplegia better preserve myocardial tissue function, as measured via left ventricular systolic and end-diastolic pressures [111]. Direct toxic effects due to individual cardioplegic solution components can also occur, as, for example, the toxicity observed with pyruvate at concentrations of 5 mmol/L, polyethylene glycol at concentrations of 5 mmol/L, Alanine-Glutamine at concentrations of 20 mmol/L, and glutathione at 3 mmol/L and dextran at 0.57 mmol/L. This toxicity can also be due to the inappropriate stimulation of cellular metabolism during hypothermic ischemia for some components [112].

**Figure 1:**
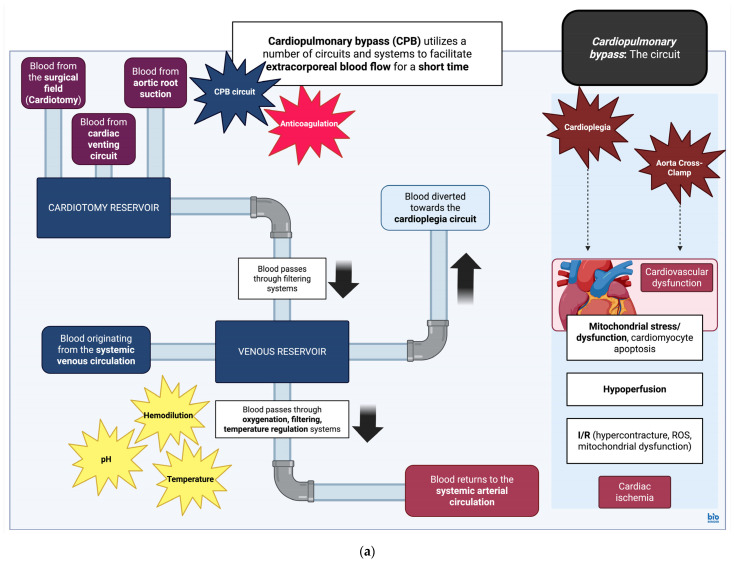

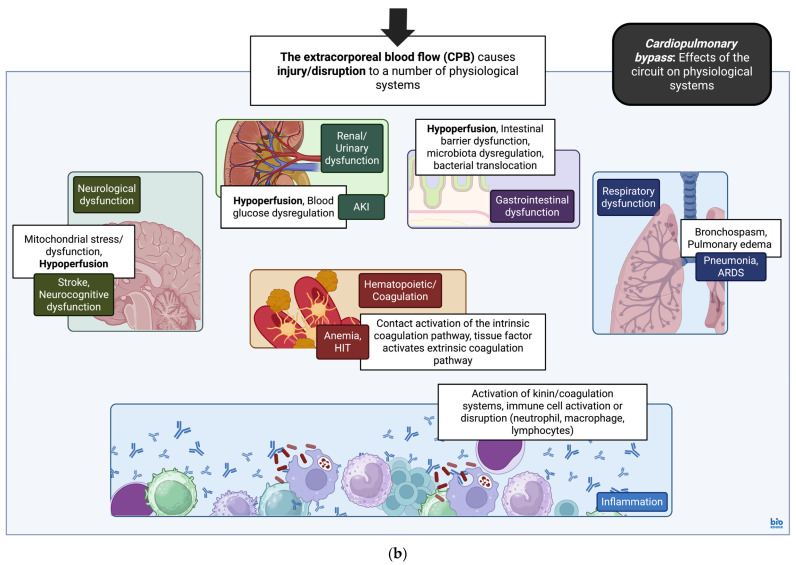
(**a**) **Cardiopulmonary bypass (CPB): the circuit.** Simplified diagram and infographic of CPB and effects of cardioplegia and aortic cross-clamp on myocardial physiology. Detrimental aspects of the circuit are noted in star text boxes. CPB, cardiopulmonary bypass; I/R, Ischemia/Reperfusion; and ROS, reactive oxygen species [106]. Created in BioRender. Stougiannou, T. (2025) https://BioRender.com/5d5ddga. (**b**) **Cardiopulmonary bypass (CPB): effects of the circuit on physiological systems.** Simplified diagram and infographic of CPB and general effects on physiological systems. Details of the CPB effects on immune physiology are included as well. CPB along with dysfunction across systems contributes to a generalized inflammatory response, with variable effects on immune cell populations. This includes upregulation of neutrophil activity, reduced expression of TLR2/4 on the surface of monocytes, and upregulation of alveolar macrophage activity. The upregulation of arginase-1 in neutrophils, in turn, causes a downregulation of CD4+ T-lymphocyte activity. Thus, during and after CPB, although there is increased production of pro-inflammatory cytokines along with neutrophil activity, immune cell functions are disrupted. AKI, acute kidney injury; ARDS, acute respiratory distress syndrome; CD4, CD4 molecule; CPB, cardiopulmonary bypass; HIT, heparin-induced thrombocytopenia; and TLR2/4, Toll-like receptor 2/4 [113,114,115,116,117,118]. Created in BioRender. Stougiannou, T. (2025) https://BioRender.com/ihjp8ih.

Finally, CPB can have detrimental effects on a number of systems, including effects on coagulation due to contact activation and the release of tissue factor from the surgical field of operation [113]. Neurological sequelae can be mainly attributed to I/R injury as well as hypoperfusion and hypoxia [114]. Hypoperfusion, along with pre-existing conditions such as T2DM or poor glucose regulation, can also contribute to acute kidney injury (AKI) [115]. Reactions to substances employed for heparin reversal (protamine) or blood products may lead to bronchospasm [116], whilst excessive fluid transfusion can cause transfusion-related acute lung injury (TRALI) and non-cardiogenic pulmonary edema [117]. Finally, the general hypoperfusion associated with the operation can also lead to the hypoperfusion of abdominal viscera and gastrointestinal injury. This eventually results in injury/cell death of the intestinal epithelium, dysregulation in intestinal microbiota, and, as a result, bacterial translocation across the intestinal barrier causing multiple organ dysfunction [118] (Figure 1).

### 4.2. Cardiopulmonary Bypass: General Overview of the Inflammatory Response During Cardiopulmonary Bypass

Many of the factors and organ system sequelae described in the previous subsection collectively induce a postoperative inflammatory response. The earliest occurrence in this sequence of events is contact of the circulating blood with the negatively charged surfaces of the CPB circuit. This contact initiates the intrinsic coagulation pathway via the activation of factor FXII and sequential cleavage of high-molecular kininogen (HMWK) into prekallikrein and, finally, kallikrein. This acts as a trigger for leukocyte recruitment [119] and other coagulation factors [120]. Factor FXII also stimulates the aggregation and degranulation of neutrophil cells. The kallikrein–kinin system factor, bradykinin, functions as chemotactic stimulus for neutrophils, leading to increased vascular permeability and contributing to the overall inflammation observed [121]. Kallikrein further feeds into this cycle by continuously stimulating factor FXII activation, HMWK cleavage into bradykinin, and plasminogen cleavage into plasmin, along with additional stimulation of alternative complement pathways [122]. The extrinsic coagulation pathway also presents a link between coagulation and the inflammatory response after CPB; the tissue factor originating from the operating field triggers activation of the coagulation factor FVII [113,122,123]. The tissue factor can also be increased in various other conditions such as DM, atherosclerosis, and acute coronary syndromes, which may further complicate these interactions as well [124]. In general, coagulation factors FVII, FX, FII (thrombin), and FI (fibrin) are all pro-inflammatory, while FII (thrombin) and tissue factor/FVII/FX, as well as tissue factor/FVII complexes, can all activate protease-activated receptors (PAR) on the surface of platelets [125]. These will then stimulate the production of pro-inflammatory signals, including TNF-α, IL-1, and IL-6 [124].

Coagulation pathways will eventually produce factor FII (thrombin), which adheres to the fibrinogen bound on the surface of the CPB circuitry [126]; in these areas, it becomes resistant to antithrombin III, the latter of which is usually activated upon administration of heparin intraoperatively [122]. There, it will contribute to the further activation of factor FII (thrombin), causing the continuous accumulation of activated platelets. While thrombi rich in factor FI (fibrin) can be limited with high doses of heparin, thrombi rich in factor FII (thrombin) cannot be as easily prevented; these factor FII (thrombin) complexes depositing on the CPB circuit will thus continue to contribute to circulating FII (thrombin) levels [127].

Fibrinogen fragments produced with thrombi dissolution can reduce the expression of vascular endothelial (VE)-cadherin and increase permeability of the endothelial barrier, contributing to inflammatory processes [128]. In addition, factor FI (fibrin) will stimulate the production of C3a and C4a [129], while factor FII (thrombin) will stimulate the expression of the platelet-adhesion factor (PAF) on the endothelial cell surface, along with P-selectin and intercellular adhesion molecule-1 (ICAM-1), facilitating neutrophil adhesion [130]. Neutrophils can also contribute to inflammation via the release of formations comprising deoxyribonucleic acid (DNA) and histone material, known as neutrophil extracellular traps (NET). These further enhance the generation of factor FII (thrombin) via factor FXII and the intrinsic coagulation pathway, as well as via toll-like receptor 2 (TLR2)/TLR4-dependent mechanisms [131]. In general, the production of NET during CPB correlates to overall CPB duration [132], while higher NET levels 3 days after surgery, in the form of histone-DNA complexes as well as double-stranded DNA, have also been associated with high risk for the occurrence of postoperative atrial fibrillation (POAF) [133].

Contant activation and stimulation of kallikrein leads to the activation of complement factors C3a and C5a; the CPB circuit surface usually lacks the appropriate inhibitors normally found on the endothelial cell surface, which would limit the activation of complement proteins [122,134]. C3a and C5a function as anaphylatoxins, with C5a correlating with postoperative myocardial injury [135] and both C3a and C5a correlating with postoperative blood loss [136]. C5a acts a chemoattractant for neutrophils via the C5aR1 receptor [137], while C3a stimulates platelet aggregation via its cognate receptor on the platelet surface, C3aR [138]. Activation of the alternative complement pathway can occur via endotoxins (LPS) released into the circulation due to splanchnic hypoperfusion and transient permeability of the intestinal epithelial barrier [139]. The LPS released within the systemic circulation as a result, will then bind to receptors on the surfaces of macrophages, forming TLR4/myeloid differentiation-2 (MD-2)/CD14 receptor complexes. Eventually, the NF-κB pathway is stimulated with the production of pro-inflammatory cytokines (TNF-α, IL-6) [140]. However, LPS can stimulate endothelial cell activation as well, with the subsequent upregulation of adhesion molecules such as E-Selectin. Activated endothelial cells, especially those that upregulate both E-selectin and vascular cell adhesion molecule 1 (VCAM-1) on their cell surface, will express various pro-inflammatory cytokines and chemokines including IL-6, IL-8, MCP-1, C-X-C motif chemokine ligand 6 (CXCL6), and CXCL10 [141]. Exposure of endothelial cells to continuous laminar flow instead of the physiological pulsatile flow can have detrimental effects as well. Continuous laminar flow stimulates NF-κB and activator protein-1 (AP-1) signaling, leading to upregulation of the pro-inflammatory cytokines TNF-α, IL-1, and IL-6 in pulmonary endothelial cells [142].

ICAM-1 adhesion molecules are upregulated on the surface of cardiomyocytes after cardioplegic arrest/CPB. These adhesion molecules serve as attachment points for circulating neutrophils, which, upon binding via neutrophil CD18/ICAM-1, lead to cardiomyocyte oxidative injury [143]. In turn, ICAM-1 expression can be upregulated by many pro-inflammatory cytokines including TNF-α, IL-1, and IL-6; cardiomyocytes also produce IL-6, an event attributed to local ischemic processes, pro-inflammatory cytokines (TNF-α) released by cardiac mast cells, as well as I/R injury and NF-κB signaling [144]. IL-6 has been produced in considerable quantities by cardiomyocytes in animal models, even with cold cardioplegic arrest, while it has also been detected in high quantities in both heparin and non-heparin coated circuits and in a wide variety of CPB settings [122]. IL-6 activates the Janus kinase (JAK)/signal transducer and activator of transcription (STAT3) signaling pathway, which regulates the immune response and interacts with NF-κB signaling pathways [143]. IL-6 may also contribute to myocardial dysfunction/’stunning’ after cardiac surgery, as it has been associated with negative inotropic effects. However, it may also provide a cardioprotective effect [145], both during and after cardioplegic arrest/CPB. This cardioprotective effect has been attributed to the prevention of cardiomyocyte apoptosis [144] along with various other anti-inflammatory effects [146].

IL-36 is another cytokine that contributes to cardiomyocyte injury during cardioplegic arrest/CPB. IL-36, via binding to its cognate receptor IL-36R, regulates the sirtuin 1 (SIRT1)/FOXO1/p53 signaling pathway in cardiomyocytes [147]. SIRT1 generally upregulates endothelial nitric oxide synthase (eNOS), facilitating vascular relaxation, while it can also regulate cellular metabolism, survival, and apoptosis [148]. In endothelial cells, SIRT1 prevents endothelial senescence via the SIRT1/eNOS pathway. IL-36 also induces Th1-lymphocyte and pro-inflammatory M1 macrophage polarization [149]. The stimulation of pro-inflammatory cell phenotypes will eventually lead to further secretion of pro-inflammatory cytokines from cardiomyocytes, including TNF-α and IL-1β. Finally, IL-36 contributes to myocardial oxidative stress via the stimulation of inducible NO synthase (iNOS) and downregulation of eNOS [147]. The inhibition of IL-36 in animal models of CPB usually prevents iNOS activation and favors the upregulation of eNOS. Furthermore, the inhibition of IL-36 also reduces the expression of chemotactic factors in cardiomyocytes (MCP-1, C-C Motif Chemokine Receptor 2 [CCR2], CXCL2, and CCL12), preventing the recruitment of pro-inflammatory circulating monocytes. Upon the absence of IL-36, signaling through the SIRT1/FOXO1/p53 pathway is upregulated, preventing cardiomyocyte cell death as well [147].

### 4.3. Cardiopulmonary Bypass: Contributions of Macrophage and T-Lymphocyte Populations to the Inflammatory Response and Immune Dysfunction with Cardiopulmonary Bypass

Though monocyte levels have not been observed to fluctuate after CPB, the surface expression of some receptors is altered, with a reduction in the expression of TLR2/TLR4; reduced expression of TLR2 has been associated with the occurrence of systemic inflammatory response syndrome (SIRS) and pneumonia in the postoperative period [150]. Downregulation of human leukocyte antigen-DR isotype (HLA-DR) also occurs after CPB, possibly contributing to the associated immunoparesis and nosocomial infections [151,152]. Despite this overall trend, alveolar macrophages are upregulated after cardiac surgery with CPB, expressing markers such as CD11a, CD11b, CD11c, and CD18. This may occur directly due to CPB-mediated activation of complement and neutrophils or due to lung I/R injury after release of the aorta cross-clamp, as well as hemodilution and rewarming after hypothermia. Activation of alveolar macrophages contributes to pyroptosis via activation of the NLR Family Pyrin Domain Containing 3 (NLRP3) inflammasome and secretion of caspase-1, which, along with IL-1β and IL-18, contribute to inflammation. NLRP3 inflammasomes in alveolar macrophages can, in turn, also be stimulated by TLR and DAMP, originating in nearby necrotic cells. The ensuing pyroptosis promotes the release of components such as high Mobility Group Box 1 (HMGB1), which further enhance lung injury in the animal models of CPB [153]. In general, CPB induces an increase in pro-inflammatory factors within the pulmonary circulation, particularly IL-6, IL-8, and TNF-α, owing to their secretion from alveolar macrophages. The resulting inflammatory response in the pulmonary circulation is generally greater compared to the systemic inflammatory response [154].

Macrophage Migration Inhibitory Factor (MIF) is a pro-inflammatory cytokine secreted by various cell types, including immune cell groups such as macrophages and other non-immune cells. MIF expression is upregulated and augmented by pro-inflammatory factors such as LPS (endotoxins), TNF-α, as well as glucose and insulin [155]. It promotes the accumulation of additional immune cell groups via the upregulation of adhesion molecules (VCAM-1, ICAM-1) on the surface of endothelial cells leading to endothelial-mediated secretion of the chemoattractant MCP-1 [156]. Furthermore, MIF also binds CXCR2 and CXCR4, contributing to the accumulation of monocytes and lymphocytes [157]. Regarding effects on the metabolism, MIF, via adenosine monophosphate (AMP)-activated protein kinase (AMPK) and under ischemic conditions, leads to an increase in ATP production, namely due to increased GLUT4 translocation to the cell membrane surface and increased Phosphofructokinase-2 (PFK-2) activity. Thus, overall activity through the glycolytic pathway in cardiomyocytes is upregulated [158]. MIF also contributes to the insulin resistance effects mediated by TNF-α due to the reduced AKT phosphorylation and, as a result, reduced phosphorylation of IRS-1, all necessary for signal transduction via the insulin receptor [159]. In general, MIF has been observed to increase after cardiac surgery primarily due to cardiomyocyte I/R injury and less so due to inflammation. It has been associated with antioxidant activity and a reduced incidence of AKI, as well as POAF in this regard [160] and inversely associated with organ dysfunction occurring after cardiac surgery [161]. However, higher circulating MIF levels, 6 h after CPB, have been clinically associated with pulmonary dysfunction and worse pulmonary outcomes after cardiac surgery [162].

Regarding lymphocyte function, this has been shown to be impaired by myeloid suppressor cells, produced from hematopoietic stem cell progenitors in response to pro-inflammatory mediators such TGF-β, IFN-γ, IL-4, IL-13, and TLR ligands. These cells are generally characterized by the increased production of ROS and reactive nitrogen species (RNS), as well as Arg-1 [163]. In particular, 24 to 72 h after cardiac surgery, an increase in Arg-1 activity is associated with downregulation in the CD3ζ component of the T-lymphocyte TCR receptor, eventually reducing the T-lymphocyte response/proliferation in response to antigenic stimulation. This increase in Arg-1 activity can be, in turn, attributed to the release of cytokines after CPB and the subsequent activation of neutrophils [164]. Additional studies with pediatric cardiac surgery patients have also associated the increased levels of pro-inflammatory cytokines postoperatively with T-lymphocyte lymphopenia; these reduced T-lymphocyte levels will increase the risk for infections in the postoperative period. This study also provides evidence for monitoring of the postoperative lymphopenia, rather than measurement of CRP or procalcitonin, as a better predictor of the occurrence of associated infections during this period [165]. Postoperative lymphocyte decreases are mainly observed in CD4+ T-lymphocyte groups, with no significant effects on CD8+ T-lymphocyte levels [166].

In general, the inflammatory response instigated during cardiac surgery with CPB stimulates platelet activation and aggregation, as has already been reiterated. Activation is evident by the upregulation of platelet glycoprotein IIb/IIIa (GpIIb/IIIa), which binds fibrinogen; GpIV, which binds thrombospondin and collagen; and finally, P-Selectin, which interacts with other cells such as endothelial, neutrophil, monocyte, and other immune cell populations [166]. In response to surgery, the increased expression of P-Selectin on the surface of activated platelets contributes to the formation of platelet-T-lymphocyte aggregates (PTCA) via P-Selectin/P-Selectin glycoprotein ligand-1 (PSGL-1) receptor interactions. PTCA formations then exhibit an inverse correlation with the declining CD4+ T-lymphocyte populations postoperatively. They are also directly associated with the rising levels of CD4+ CD25+ forkhead box P3 (FOXP3)+ and CD8+ CD25+ FOXP3+ Treg-lymphocytes postoperatively [166], attributed to the platelet-mediated release of TGF-β1 [167]. Thus, overall, PTCA formation facilitates the binding of CD4+ T-lymphocytes, their migration into lymph nodes, and the emergence of Treg-lymphocyte types, accounting for the alterations in T-lymphocyte populations after cardiac surgery [166].

## 5. Cardiopulmonary Bypass (CPB) and Its Effects on the Inflammatory State in Type 2 Diabetes Mellitus

As it has already been extensively described, T2DM is a condition characterized by chronic hyperglycemia, insulin resistance, disruptions in lipid metabolism, and low-grade inflammation with its many sequelae [168]. CPB can also be accompanied by a decrease in insulin levels; in simulated conditions of extracorporeal circulation, this decrease has been attributed to the degradation of insulin. This degradation, in turn, is due to circulating products of hemolysis and, to a lesser extent, due to the adherence of insulin on circuit surfaces [169]. The development of insulin resistance during cardiac surgery with CPB has also been attributed to the developing inflammatory reaction, which can, in turn, lead to insulin resistance [170]. In a study of infants undergoing cardiac surgery with CPB, for example, an inverse relationship between IL-6 and TNF-α levels (signifying inflammation) and adiponectin has been observed. Reduction in adiponectin levels, in turn, contributes to insulin resistance [170]. During surgery, there is also secretion of pro-inflammatory cytokines from both subcutaneous and epicardial adipose tissue. This includes IL-6, TNF-α, CD45, resistin, and MCP-1 all secreted from subcutaneous adipose tissues and IL-6, resistin, and MCP-1 released from epicardial adipose tissues [171]. Regarding the cytokine IL-6, increased levels have also been identified in the peripheral blood of animal models [172]. All these inflammatory factors contribute to the development of insulin resistance in the perioperative period, even in the absence of preoperative DM [171]. Despite this association, however, some clinical trials point to no association between insulin and the pro-inflammatory (IL-6, TNF-α) versus anti-inflammatory (IL-10) cytokine balance in the postoperative period [173]. Conversely, other studies associate hyperglycemia with potentiation of the inflammatory response [174,175], an effect which could be possibly attributed to the unique immune conditions generated by CPB, i.e., a combination of pro-inflammatory conditions with the dysfunction of certain immune cell groups.

The inflammatory reaction occurring during CPB can also be associated with deleterious sequelae in endothelial function and endothelial permeability in DM [176]. More specifically, hypoxia induces the upregulation of factors such as HIF-1α, in turn leading to an upregulation of VEGF, a growth factor involved in angiogenesis and wound healing. It is also frequently upregulated under pro-inflammatory conditions [177]. While variations in VEGF levels across DM groups are not significant, on the other hand, VEGF levels become considerably elevated in DM patients after cardiac surgery with CPB, even 4 days after the event. Similarly, hepatocyte growth factor (HGF) levels are considerably increased as well [176]. All these factors are thought to be upregulated as part of a signaling pathway active during hypoxia, stimulated by HIF-1α. In response to hypoxia during the operation, HIF-1α expression, along with other proteins of the HIF-1α pathway (cyclic AMP response element binding protein [CREB], E1A binding protein p300 [EP300]) are increased significantly. In turn, this increase in VEGF and HGF expression modulates endothelial permeability, further stimulating leukocyte extravasation and contributing to inflammation [176]. CPB may also have a greater effect on vascular permeability in T2DM, as seen with the upregulation of VE-Cadherin phosphorylation and the degradation of β- and γ-catenin in patient cohorts characterized by poor DM control. Though phosphorylation in these proteins is generally induced postoperatively after CPB in all groups, it is augmented in patients with poor DM control. In general, VE-Cadherin, its association with β-and γ-catenins, and the actin cytoskeleton are all regulated by VEGF [178,179]. As a result, as endothelial cell-to-cell junctions are disrupted, endothelial dysfunction, along with increases in endothelial permeability, ensues [180].

Cardiac surgery with CPB also affects the biological process of autophagy. Autophagy is normally involved in the degradation and recycling of cellular proteins; this process is impaired in myocardial tissues in patients with diabetic cardiomyopathy due to disruption in the cardiomyocyte SIRT3/forkhead box O3α (FOXO3α) signaling cascade [181]. This eventually results in the downregulation of autophagy in cardiomyocytes, leading to cardiomyocyte apoptosis [182]. FOXO3α also reduces oxidative stress within cardiomyocytes under physiological conditions; therefore, downregulation of FOXO3α is associated with the augmentation of oxidative stress instead [183]. After cardiac surgery with CPB, there is physiological upregulation in FOXO3α, an observation in line with the contribution of this factor in pathways aimed at the removal of dysfunctional organelles due to inflammation and oxidative stress. In DM, a paradoxical decrease is observed instead [181]. Thus, while one would expect the hypoxic, I/R, inflammatory, and metabolic insults associated with CPB to normally upregulate enzymes such as SIRT1, the transcription factor FOXO3α, and the peroxisome proliferator-activated receptor gamma coactivator 1a (PGC1a) enzyme, involved in autophagy (SIRT1, FOXO3α) and fatty acid oxidation (PGC1a), these responses are all blunted in DM. Activation of autophagy pathways is prevented and the intracellular damage cannot be restricted, contributing to cardiomyocyte dysfunction and apoptosis [181]. Reduced levels of PGC1a postoperatively have been associated with adverse postoperative outcomes including new onset POAF, observed in DM cohorts [184]. In addition, impairment of cardiomyocyte autophagy due to impairment in the processing of enzymes participating in the autophagy pathway, including microtubule associated protein 1 light chain 3 beta (LC3B), which participates in the formation of autophagosomes, have also been associated with POAF. This is due to the ultrastructural atrial remodeling occurring as a result of impairment in cardiomyocyte autophagy pathways [185].

Often, patients with DM exhibit higher rates of morbidity after cardiac surgery with CPB. The I/R injury normally incurred during cardioplegic arrest with CPB exacerbates injury in systems already vulnerable to injury due to pre-existing disease. As expected, in animal models of T2DM, when subjected to CPB, the myocardium is highly vulnerable to I/R injury, with evidence of increased lipid deposition, myocardial fiber disruption, and cardiomyocyte death. In these models, inflammation, triggered by ROS and caspase-1-mediated pyroptosis, further contributes to tissue insult as well [168]. Diabetic cardiomyopathy is frequently associated with postoperative disruptions in the expression of mitogen kinase enzymes; this can impact and augment microvascular dysfunction in DM [186]. Microvascular reactivity, in particular, is impaired with poorly controlled DM; in studies examining postoperative microvascular reactivity, measurement of the vasodilatory response to substances such as adenosine diphosphate (ADP) and substance P has been carried out [187]. The PKC isoforms, PKC-α and PKC-β, are also upregulated in endothelial cells and VSMC with poorly controlled DM in tissues such as skeletal muscle. This is due to the hyperglycemia of poorly controlled DM, leading to the generation of AGE compounds; however, ROS are also a trigger for the upregulation of these PKC isoforms [188]. Activation of these enzymes in skeletal muscle arterioles will contribute to the dysfunctional arteriolar response after cardiac surgery with CPB in cases of poorly controlled DM. In essence, the physiological vasodilation that would be expected upon administration of the test compound, is prevented.

Both PKC-α and PKC-β isoforms potentiate VSMC vasoconstriction via the stimulation of Cyclooxygenase-2 (COX-2)-mediated production of Prostaglandin H_2_ (PGH_2_) and Thromboxane A_2_ (TXA_2_) [188]. COX-2 levels have been shown to be augmented after cardiac surgery with CPB. In this setting, however, these elevations could be attributed to the general inflammatory response, as COX-2 has been shown to be increased across all patient groups. However, this increase is further augmented in groups with poor DM control [189]. In these same patient cohorts, vascular reactivity is disproportionately disrupted, as made evident by studies evaluating the response of cardiac microvessels to TXA_2_ [186] and ADP [180]. In addition, PKC contributes to the generation of ROS via NOX activation [188]. In relevant studies, levels of PKC-α and PKC-β, along with levels of protein oxidation and nitrotyrosine species, are all significantly elevated after cardiac surgery with poorly controlled DM; this leads to endothelial dysfunction [187]. Finally, PKC is involved in VEGF production [188]; this could offer an explanation for the increase in VEGF occurring in DM patients after cardiac surgery with CPB as well [176] (Table 4, Table 5 and Table 6).

Another phenomenon often associated with cardiac surgery is low cardiac output syndrome (LCOS), referring to postoperative cardiac dysfunction that results in reduced cardiac output. It is most commonly defined as a cardiac index (CI) < 2.0 L/min/m^2^, ionotropic support applied for more than 30 min to achieve systolic blood pressures greater than 90 mmHg or CI < 2.2 L/min/m^2^, use of one or more inotropic medications, and lactate levels greater than 2.0 mmol/L, or finally, the use of IABP or mechanical ventricular assistance [190]. Many mechanisms can contribute to this clinical presentation, including myocardial dysfunction owing to the I/R-mediated Ca^2+^ dysregulation within cardiomyocytes. These include the decreased uptake of Ca^2+^ by ATPase Sarcoplasmic/Endoplasmic Reticulum Ca^2+^ Transporting 2 (SERCA2a) receptors in the sarcoplasmic reticulum and increased leak of Ca^2+^ from Ryanodine Receptor 2 (RYR2). Eventually this causes mitochondrial damage due to the upregulation of MPTP and Na^+^/Ca^2+^ exchanger activity, as well as activation of inflammatory pathways, triggering cellular apoptosis [191]. LCOS has been described as a form of acute heart failure after cardiac surgery, although it differs from heart failure in aspects of etiology, as the development of LCOS is influenced by cardiac surgery [192]. Furthermore, though overactivation of autophagic pathways have been implicated in I/R in the context of heart failure progression, via overexpression of the protein Beclin-1 contributing to increased injury, the specific molecular pathways connecting LCOS after cardiac surgery with autophagy have not been directly explored [193]. It is well-known, however, that the postoperative myocardial dysfunction characterizing LCOS is augmented in DM; in addition, DM in cardiac surgery is associated with higher early and late postoperative mortality [194]. Finally, both POAF and LCOS have been associated with increased early and late postoperative mortality, as well as increased length of hospital and ICU stay in cohorts of patients undergoing cardiac surgery [195,196,197].

**Table 4:** Effects of cardiopulmonary bypass (CPB) on inflammatory and immune parameters associated with diabetes mellitus (DM). Effects described are observed in cardiac surgery with CPB in relation to the presence of DM, unless otherwise noted. CD45, CD45 Molecule; CPB, cardiopulmonary bypass; CREB, Cyclic Adenosine Monophosphate (cAMP) response element-binding protein; CREB5, Cyclic Adenosine Monophosphate (cAMP) response element-binding protein 5; DM, diabetes mellitus; EP300, E1A binding protein p300; HGF, hepatocyte growth factor; HIF-1α, hypoxia inducible factor 1-alpha; IL-1β, Interleukin-1β; IL-6, Interleukin-6; IL-8, Interleukin-8; IRS-1, insulin receptor substrate-1; ITA, internal thoracic artery; MCP-1, Monocyte Chemoattractant Protein-1; MYC, Myelocytomatosis Oncogene; TNF-α, tumor necrosis factor alpha; and VEGF, vascular endothelial growth factor.

Study (Reference)	Description	Model	Immune Effects of Cardiac Surgery with CPB
Antunes et al., 1997 [198]	Increased risk for postoperative wound infections, mediastinitis; risk augmented with bilateral ITA harvesting	Human	Mediastinitis (Increase)
Matata and Galiñanes, 2000 [199]	Increase in pro-inflammatory factors and cytokines; increase in complement factors (C3a) greater and more immediate, activation of immune cells (neutrophils) and secretion of elastase persists for longer periods after CPB	Human	Qualitative differences in inflammatory reaction in DM versus non-DM cohorts
Groom et al., 2004 [200]	DM, temperature-interacting variables in infectious mediastinitis; rates of mediastinitis increase from 0.7% (lower than 37° C) up to 3.3% (greater than 38 °C); no such effect observed in non-DM groups	Human	Mediastinitis (Increase)
Voisine et al., 2004 [201]	Upregulation of transcriptional activators related to inflammation (MYC, IL-8, IL-1β, VEGF, amphiregulin, and IRS-1)	Human	Pro-inflammatory gene expression (Increase)
Kremen et al., 2006 [171]	Increase in pro-inflammatory cytokine secretion from subcutaneous (IL-6, TNF-α, CD45, resistin, and MCP-1), epicardial adipose tissue (IL-6, resistin, and MCP-1); association of pro-inflammatory factor secretion with insulin resistance up to 24 h after CPB	Human	Pro-inflammatory factors (Increase)
de Lange et al., 2007 [172]	Higher levels of IL-6 in animals with DM compared to non-DM groups	Rat	Pro-inflammatory factors (Increase)
Emani et al., 2009 [176]	Higher levels and larger increase in circulating pro-inflammatory cytokines (VEGF, HGF) with increased expression of pro-inflammatory factors HIF-1α, CREB, and EP300 in DM compared to non-DM groups	Human	Pro-inflammatory cytokines (Increase), pro-inflammatory gene expression (Increase)
Zakrzewski et al., 2010 [202]	No association of the postoperative inflammatory response with renal failure	Human	N/A
Le Guillou et al., 2012 [203]	Higher levels and larger increase in TNF-α levels DM groups	Rat	Pro-inflammatory cytokines (Increase)
Zhou et al., 2024 [168]	Higher levels and larger increase in IL-6, TNF-α, inflammation, and caspase-1-mediated pyroptosis in T2DM groups (cardiac muscle)	Rat	Pro-inflammatory cytokines (Increase)

**Table 5:** Effects of cardiopulmonary bypass (CPB) on postoperative vascular reactivity associated with diabetes mellitus (DM). Effects described are observed in cardiac surgery with CPB in relation to the presence of DM, unless otherwise noted. ADP, Adenosine Diphosphate; COX-1/2, Cyclooxygen-ase-1/2; CPB, cardiopulmonary bypass; DM, diabetes mellitus; ERK1/2, extracellular signal-related kinase 1/2; JNK, c-Jun N-terminal kinase; MKP-1, mitogen-activated protein kinase phosphatase-1; PKC-α/β, protein kinase C alpha/beta; TXA2, Thromboxane A2; VE-Cadherin, vascular endothelial cadherin; and p38-MAPK, mitogen-associated protein kinase 14.

Study (Reference)	Description	Location	Model	Vascular Response to Cardiac Surgery with CPB
Feng et al., 2012 [187]	Arteriolar response to vasodilatory substances (ADP, substance P) impaired in poorly controlled DM; Increased levels of PKC-α, PKC-β	Skeletal muscle microvessels	Human	Response to vasodilation (Decrease)
Le Guillou et al., 2012 [203]	Arteriolar response to vasoconstrictor substances (phenylephrine) is enhanced, while response to vasodilatory substances (acetylcholine) is impaired	Mesentery microvessels	Rat	Response to vasodilation (Decrease), response to vasoconstriction (Increase)
Feng et al., 2013 [186]	Arteriolar response to vasoconstrictor substances (TXA_2_) impaired in all patients, effect augmented in patients with poorly controlled DM	Cardiac muscle, coronary microvessels	Human	Response to vasoconstriction (Decrease)
	Changes in protein kinase expression, including p38-MAPK (decrease), ERK1/2 (decrease), and JNK (increase); MKP-1 levels higher in poorly controlled DM with no significant changes due to CPB	Cardiac muscle		Alterations in levels of MAPK, ERK, and JNK kinase activity
Feng et al., 2016 [180]	Arteriolar response to vasodilatory substances (ADP) impaired in poorly controlled DM	Cardiac muscle, coronary microvessels	Human	Response to vasodilation (Decrease)
	Higher levels of phosphorylated endothelial VE-Cadherin in poorly controlled DM; higher rates of VE-Cadherin, β-, γ-catenin degradation with endothelial cell-to-cell junction disruption in poorly controlled DM			Adherens-junction activation/localization in coronary endothelial cells (Decrease)
Feng et al., 2017 [189]	Arteriolar response to vasodilatory substances (bradykinin) impaired in poorly controlled DM; higher levels and larger increase in COX-2 in poorly controlled DM; no effects on COX-1 expression	Skeletal muscle microvessels	Human	Response to vasodilation (Decrease)

**Table 6:** Effects of cardiopulmonary bypass (CPB) on postoperative oxidative stress and other parameters associated with diabetes mellitus (DM). Effects described are observed in cardiac surgery with CPB in relation to the presence of DM, unless otherwise noted. Ang1, Angiopoietin 1; BCM-xL, B-cell lymphoma-extra-large; CAD, coronary artery disease; CPB, cardiopulmonary bypass; CREB5, Cyclic Adenosine Monophosphate (cAMP) response element-binding protein 5; DM, diabetes mellitus; FOXO3α, Forkhead Box O3α; GPX4, Glutathione Peroxidase 4; MWM, Morris water maze; MYC, Myelocytomatosis Oncogene; NOX4, Nicotinamide Adenine Dinucleotide Phosphate (NADPH) Oxidase 4; NT3, Neurotrophin-3; ORAC, oxygen radical absorbing capacity; PGC-1α, Peroxisome Proliferator Activated Receptor Gamma (PPARG) Coactivator 1 Alpha; SIRT1, Sirtuins 1; SLC25A40, Solute Carrier Family 25, Member 40; and TGF-β, transforming growth factor beta.

Study (Reference)	Description	Model	Effects of Cardiac Surgery with CPB on Oxidative Stress
Matata and Galiñanes, 2000 [199]	Higher levels and larger increase in lipid hydroperoxides, protein carbonyls, and NOX4 proteins in DM groups	Human	Increase
Doenst et al., 2005 [204]	Hyperglycemia is an independent risk factor for mortality regardless of DM due to mechanisms involving immune cells (monocyte, neutrophil), endothelial function, and the pro-inflammatory state; association with insulin resistance and other comorbidities (CAD, diabetic cardiomyopathy)	Human	N/A
de Lange et al., 2007 [172]	No differences in neurocognitive performance tests (MWM) in DM versus non-DM groups	Rat	N/A
Marty et al., 2008 [205]	Increase in ascorbyl radical/vitamin C ratios, decrease in ORAC values in DM; reduced capacity for the neutralization of oxidative stress	Human	Increase
Feng et al., 2012 [187]	Increased levels of protein oxidation in skeletal muscle, contribution to levels of protein oxidation by cardiac surgery with CPB not significant	Human	No significant effect
Cao et al., 2013 [170]	Reduction in adiponectin due to inflammation contributes to perioperative insulin resistance up to 48 h after CPB	Human	N/A
Mahmood et al., 2019 [206]	Baseline levels of PGC-1α reduced in DM preoperatively, decrease is further augmented postoperatively; reduced levels of antioxidant (NOX4, GPX4), angiogenic (TGF-β, NT3, Ang1), and anti-apoptotic (BCM-xL) factors in DM postoperatively; downregulation of proteins involved in mitochondrial energy production (CREB5, SLC25A40) and angiogenesis	Human	Impaired mitochondrial function and beta-oxidation
Zhang et al., 2021 [181]	Absence of the physiological postoperative FOXO3α response, alterations in levels of effectors involved in mitochondrial FA β-oxidation (PGC-1α) and autophagy (SIRT1, FOXO3α), and downregulation of autophagy in cardiac tissue	Human	Paradoxical decrease in FOXO3α, absence of physiological upregulation in SIRT1 and PGC-1α
	No explicit association between upregulation of autophagy/mitochondrial biogenesis and improved clinical outcomes in this study		
Snel et al., 2024 [207]	Increase in ketone bodies throughout cardiac surgery, peak observed at the end of aortic cross-clamp; no differences in ketone body concentrations based on T2DM status	Human	No significant effect
Zhou et al., 2024 [168]	Levels of lipid deposition, disruption of myocardial fiber architecture, and cardiomyocyte apoptosis increased in T2DM groups	Rat	Increase

## 6. Conclusions

T2DM is a disease characterized by insulin resistance and chronic inflammation affecting the structure and function across cardiovascular systems; immune cell functions can also be impaired in T2DM, predisposing patients to infectious complications. On the other hand, cardiac surgery with CPB is an event also characterized by an intense inflammatory response along with deleterious effects on immune cell function. Though the release of pro-inflammatory cytokines is augmented, along with the pro-inflammatory alveolar macrophage activity, immune functions in other cell groups can be negatively affected. In addition, insulin resistance, the primary pathophysiologic disturbance in T2DM, instigated by multiple factors including inflammation, can also occur after cardiac surgery with CPB, even in patients without pre-existing T2DM. As expected, due to these mechanisms, many studies in both animal models and human patients identify a cumulative effect of both cardiac surgery with CPB and DM in the oxidative stress, inflammation, and microvascular dysfunction that results.

While many of these studies involve measurements of pro-inflammatory factors in association with surgical events, additional investigations evaluating different factors participating in the same cellular pathway, with the possible modulation of gene expression or measurement of effects resulting from exogenously administrated substances in relevant animal models, could perhaps yield additional information. This could help make more specific observations about pathophysiologic pathways contributing to tissue injury after cardiac surgery. Further studies evaluating the effect of obesity-associated inflammation in DM after cardiac surgery with CPB in both animal models and human patients could help identify potential interactions between these two pro-inflammatory conditions on tissue injury and dysfunction. Other components that have been associated with both DM and surgical operations, such as tissue factor, may contribute to these complex interactions observed between cardiac surgery with CPB and DM as well. Thus, further research exploring these connections could also yield valuable information. Regarding the activation or disruption of specific immune cell populations, additional studies could be organized to evaluate the role of intrinsic cardiac tissue macrophages in response to cardioplegic arrest. This query could help us better understand how these populations contribute or prevent inflammatory responses postoperatively. Finally, T-lymphocytes have been shown to be reduced postoperatively after cardiac surgery with CPB. It could be interesting to carry out further research in order to further evaluate specific subpopulations and their effect, if any, on the I/R myocardial injury after cardioplegic arrest during the procedure.

It is therefore evident that cardiac surgery with CPB drives an already vulnerable system, characterized by poor glycemic control and the associated pathophysiological sequelae of T2DM, towards additional injury. Thus, it is with a combination of further research into the basic cellular and physiologic mechanisms that contribute to injury in T2DM cohorts, as well as clinical studies in patients undergoing cardiac surgery procedures, that better and more informed decisions can be eventually made regarding the best course of action.

## Figures and Tables

**Table 1:** Classification of diabetes mellitus along with associations with heart disease for each type. BMI, body mass index; CAN, cardiac autoimmune neuropathy; CAD, coronary artery disease; DM, diabetes mellitus; GDM, gestational diabetes mellitus; MI, myocardial infarction; MODY, maturity-onset diabetes mellitus; NAFLD, non-alcoholic fatty liver disease; NHNES, national health and nutritional examination survey; PAD, peripheral artery disease; PTDM, post transplantation diabetes mellitus; T1DM, type 1 diabetes mellitus; T2DM, type 2 Diabetes Mellitus; and T3DM, pancreatic diabetes mellitus or type 3c diabetes mellitus.

Classification	Cardiovascular Associations	References
T1DM	CAD (disease is more diffuse, concentric compared to T2DM), autoimmune myocarditis post MI; diabetic cardiomyopathy; CAN (cardiac arrythmia, sudden death, and no strong direct associations with increased cardiovascular risk); cerebrovascular disease; PAD; risk factors affect men, women equally	[13,15,16,17,18]
T2DM	CAD; diabetic cardiomyopathy; cerebrovascular disease; PAD; risk factor burden for T2DM diagnosis higher in women, men diagnosed at lower BMI and age, compared to women; NAFLD in association with metabolic syndrome increases risk for both T2DM and cardiovascular disease; T2DM and CKD both independent risk factors for major adverse cardiovascular events in patients with known cardiovascular disease	[9,10,18,19,20,21,22]
T3DM	CAD	[23,24]
PTDM	CAD (MI risk higher in PTDM [relative risk of 1.6 compared to risk in pre-existing DM of 1.1]); cardiac arrhythmia; cerebrovascular disease	[25,26,27]
Monogenic DM	CAD; heart failure; cerebrovascular disease; all adverse cardiovascular events (MODY) show lower incidence compared to T2DM and higher incidence compared to T1DM in a prospective cohort study of 26,198 patients in the UK	[28]
GDM	CAD; heart failure; cerebrovascular disease; association with CAD and heart failure stronger compared to cerebrovascular disease in one national cohort study of 12,025 patients (NHNES), partly attributable to T2DM; increased risk of cardiovascular complications later in life (increased risk of ~ 23%) in embryos that develop within a hyperglycemic environment	[29,30]

**Table 2:** Inflammatory mechanisms contributing to the generalized inflammatory state in T2DM. The table includes the responses of macrophage and T-lymphocyte populations to the mechanisms described in the second column. CHOP, C/EBP Homologous Protein or Growth Arrest and DNA Damage-inducible Protein 153 (GADD153); ER, endoplasmic reticulum; ERK, extracellular signal-related kinase; FA, fatty acid; FFA, free fatty acid; FOXC2, Forkhead Box C2; IFN-γ, interferon gamma; IL-13, Interleukin-13; IL-1β, Interleukin-1β; IL-4, Interleukin-4; IL-6, Interleukin-6; MCP-1, Monocyte Chemoattractant Protein-1; NF-κB, Nuclear Factor Kappa B Subunit 1; NLRP3, NLR Family Pyrin Domain Containing 3; PAI-1, plasminogen activator inhibitor 1; ROS, reactive oxygen species; T2DM, Type 2 Diabetes Mellitus; TAG, Triacylglycerol; TLR4, Toll-like receptor 4; and TNF-α, tumor necrosis factor alpha.

Mediator	Description	Immune Cell Response	References
FFA	Accumulation of TAG in adipose tissue causing accumulation of FFA in the ER; maladaptive UPR response and ER stress trigger NF-κB signaling/NLRP3 inflammasome assembly (IL-1β, IL-18)	Upregulation of M1 macrophage polarization	[45,46,55]
Lipid metabolites	Ceramides (derived from sphingolipid metabolism, TLR4 receptor activation, TNF-α, IL-1β, and IL-6 signaling) accumulate (liver, skeletal muscle); ROS production and chronic oxidative stress contribute to inflammation via NF-κB signaling/NLRP3 inflammasome assembly	Upregulation of M1 macrophage polarization	[56,57]
		Downregulation of M2 macrophage polarization	
Saturated FA	Palmitic acid leads to activation of TLR4/ERK/FOXC2 signaling, immune cell activation	Upregulation of M1 macrophage polarization	[58]
CHOP	Chaperone protein activation by ER stress, downregulation of anti-inflammatory Th2-lymphocyte cytokine secretion (IL-4, IL-13)	Downregulation of M2 macrophage polarization	[45]
Adipocyte stress	Release of MCP-1, prevention of adipocyte differentiation due to IL-13 downregulation	Upregulation of pro-inflammatory circulating monocyte and T-lymphocyte accumulation (MCP-1)	[45,46]
		M1 macrophage polarization	
Adipose tissue	Pro-inflammatory mediators (TNF-α, IL-6, ROS) reduce adiponectin secretion with disruption of FA oxidation, glucose uptake (muscle), and physiological gluconeogenesis suppression (liver) processes	Downregulation of M2 macrophage polarization	[59]
M1 macrophage	Secretion of pro-inflammatory mediators (IL-1β, IL-6, TNF-α, MCP-1, and PAI-1)	N/A	[45,46]
T-lymphocyte	General accumulation of Th1-, γδT-, Th17, and CD8+ T-lymphocyte types; downregulation of groups that attenuate/regulate immune responses (NK cells, Th2-, and Treg-lymphocyte)	N/A	[54,60]
Th1-lymphocyte	Secretion of pro-inflammatory mediators (IFN-γ), induce expression of pro-inflammatory mediators in adipose tissue (TNF-α, MCP-1)	Upregulation of pro-inflammatory circulating monocyte accumulation (MCP-1)	[53]

**Table 3:** Inflammatory mechanisms contributing to cardiovascular disease in T2DM with T2DM disease associations. The table includes the responses of macrophage and T-lymphocyte populations to the mechanisms described in the third column. 8-iso-PGF2α, 8-iso-prostaglandin F2α; AGE, advanced glycation end-products; CF, cardiac fibroblast; DAG, Diacylglycerol; DAMP, danger-associated molecular patterns; ER, endoplasmic reticulum; ERK, extracellular signal-related kinase; FOXC2, Forkhead Box C2; GLUT4, glucose transporter 4; IFN-γ, interferon gamma; IL-1β, Interleukin-1β; IL-4, Interleukin-4; IL-6, Interleukin-6; LDL, low-density lipoprotein; LPS, Lipopolysaccharide; MCP-1, Monocyte Chemoattractant Protein-1; MMP, matrix metalloproteinase; NF-κB, Nuclear Factor Kappa B Subunit 1; NLRP3, NLR Family Pyrin Domain Containing 3; NO, nitric oxide; NOX1, Nicotinamide Adenine Dinucleotide Phosphate (NADPH) Oxidase 1; NOX2, Nicotinamide Adenine Dinucleotide Phosphate (NADPH) Oxidase 2; NOX4, Nicotinamide Adenine Dinucleotide Phosphate (NADPH) Oxidase 4; RAAS, renin–angiotensin–aldosterone system; RAGE, receptor for advanced glycation end-products; ROS, reactive oxygen species; SGK1, Serum and Glucocorticoid-Regulated Kinase 1; STAT3, Signal Transducer and Activator of Transcription 3; T2DM, Type 2 Diabetes Mellitus; TF, tissue factor; TLR4, Toll-like receptor 4; TNF-α, tumor necrosis factor alpha; VSMC, vascular smooth muscle cells; and eNOS, endothelial nitric oxide synthase.

Mediator	Mechanism	Description with Cardiovascular Disease Association	Immune Cell Response	References
Cardiomyocyte	A	ER stress, hyperglycemia-mediated oxidative stress both lead to caspase-8 and caspase-9 activation triggering caspase-3 activation, cell death with release of DAMP; ROS stimulate NLRP3 inflammasome, release of TNF-α, IL-1β, and MCP-1	Upregulation of pro-inflammatory circulating monocyte accumulation (MCP-1)	[77,78]
		Diabetic cardiomyopathy		
	G	Inflammation (TNF-α, IL-6, TNF-α, IFN-γ, and LPS) and oxidative stress (NOX2, NOX4, and ROS) contribute to cardiomyocyte hypertrophy	Upregulation of M1 macrophage polarization	[71,79]
		Diabetic cardiomyopathy		
CF	E	RAAS axis activation and SGK1/STAT3 signaling, RAAS-mediated activation of NF-κB signaling with secretion of TNF-α, MCP-1, IL-6, and IL-8	Upregulation of M1 macrophage polarization	[78,80]
		Diabetic cardiomyopathy		
LDL	B	Modified LDL (glycation, oxidation) interacts with subendothelial proteoglycan, leads to macrophage-mediated phagocytosis of LDL with upregulation of pro-inflammatory IL-1β, IL-6, TNF-α, and MMP	Upregulation of M1 macrophage polarization	[68]
		Atherosclerosis, diabetic macrovascular disease		
M1 macrophage	B	Increased atherosclerotic plaque instability due to increased M1 macrophage polarization, VSMC senescence	N/A	[58]
		Atherosclerosis, diabetic macrovascular disease		
M1 macrophage	E	IL-1β produced by M1 macrophages stimulates cardiac fibroblasts	N/A	[71]
		Diabetic cardiomyopathy		
Mitochondria	C	Increased FA oxidation with cardiomyocyte hypoxia, increased production of lipid intermediaries (DAG, acylcarnitine, and ceramide) triggering ROS production, ER stress	Upregulation of M1 macrophage polarization	[56,57,81]
		Diabetic cardiomyopathy, cardiac lipotoxicity		
Platelets	F	Lipid peroxidation generates F2-isoprostanes (8-iso-PGF_2α_) due to low-grade inflammation, enhances platelet aggregation	Upregulation of M1 macrophage polarization	[82,83]
		Atherosclerosis, diabetic macrovascular disease		
ROS	D	Increased NOX activity, absence of compensatory low-level NO production (dysfunctional eNOS)	Upregulation of M1 macrophage polarization, downregulation of M2 macrophage polarization	[84,85]
		Diabetic macrovascular disease		
Saturated FA	B	High levels of palmitic acid in T2DM trigger TLR4/ERK/FOXC2 signaling	Upregulation of M1 macrophage polarization	[58]
		Atherosclerosis, diabetic macrovascular disease		
TF	F	TF upregulation in endothelial cells and circulating monocytes due to low-grade inflammation (TNF-α, IL-6)	N/A	[86]
		Atherosclerosis, diabetic macrovascular disease		
Th1-lymphocyte	E	Integrin-α4 cell-to-cell interactions with cardiac fibroblasts, transdifferentiation into myofibroblasts	N/A	[75]
		Diabetic cardiomyopathy		
Treg-lymphocyte	E	Reduced levels of Treg-lymphocytes in T2DM augment cardiac fibrosis/hypertrophy	Upregulation of M1 macrophage polarization, downregulation of M2 macrophage polarization	[76,87]
		Diabetic cardiomyopathy		

Note: Mechanisms described in the Table: Apoptosis = A, Atherosclerosis = B, cellular metabolism = C, endothelial dysfunction = D, fibrosis = E, hypercoagulative state = F, and hypertrophy = G.

## Data Availability

No new data were created or analyzed in this study.

## Abbreviations

The following abbreviations are used in this manuscript:

AdipoR1	Adiponectin receptor-1
ADP	Adenosine Diphosphate
AGE	Advanced glycation end-products
AKI	Acute kidney injury
AKT	Protein kinase B
AMP	Adenosine Monophosphate
AMPK	AMP-activated protein kinase
AP-1	Activator Protein-1
Arg-1	Arginase-1
ATP	Adenosine triphosphate
C3aR	Complement C3a receptor
C5aR1	Complement C5a receptor 1
Ca^2+^	Calcium
cAMP	Cyclic adenosine monophosphate
CCR2	C-C Motif Chemokine Receptor 2
CHOP	C/EBP homologous protein or growth arrest and DNA damage-inducible protein 153 (GADD153)
CI	Cardiac index
CK-MB	Creatine kinase-MB
CPB	Cardiopulmonary bypass
CR3	Complement receptor 3
CRP	C-Reactive protein
CTLA-4	Cytotoxic T-lymphocyte–associated protein 4
CXCL1/2/6/8A/9/10/12	C-X-C motif chemokine ligand 12
CXCR2/3/4/7	C-X-C motif chemokine receptor 2/3/4/7
DAMP	Damage-associated molecular patterns
DM	Diabetes mellitus
DNA	Deoxyribonucleic acid
ECM	Extracellular matrix
eGFR	estimated Glomerular filtration rate
eNOS	Endothelial nitric oxide synthase
ER	Endoplasmic reticulum
ERK	Extracellular signal-related kinase
FFA	free fatty acid
FOX	Forkhead box
FOXO	Forkhead box O
FOXO1	Forkhead box O1
FOXP3	Forkhead box P3
G6Pase	Glucose-6-phosphatase
*GCK*	Glucokinase
GDM	Gestational diabetes mellitus
GLUT1/2/4	Glucose transporter 1/2/4
GpIIb/IIIa/IV	Glycoprotein IIb/IIIa/IV
GSK3/3β	Glycogen synthase kinase 3/3 beta
HbA1c	Glycated hemoglobin A
HCV	Hepatitis C virus
HDL	High density lipoprotein
HDL-C	High density lipoprotein-C
HIF-1α	Hypoxia inducible factor 1 alpha
HIT	Heparin induced thrombocytopenia
HLA-DR	Human leukocyte antigen-DR isotype
HMGB1	High Mobility Group Box 1
HMWK	High-molecular-weight kininogen
HTK	Histidine, tryptophan and ketoglutarate
I/R	Ischemia/Reperfusion injury
IABP	Intra-aortic balloon pump
ICAM-1	Intercellular adhesion molecule-1
IFN-γ	Interferon gamma
IGF1-R	Insulin growth factor 1-receptor
IL-1β/4/5/6/8/9/10/12/13/17A/18/21/22/23/36	Interleukin-1β/4/5/6/8/9/10/12/13/17A/18/21/22/23/36
IL-6/9R	Interleukin-6/9 receptor
iNOS	Inducible nitric oxide synthase
IRS-1	Insulin receptor substrate-1
JAK1/3	Janus kinase 1/3
JNK	C-Jun N-terminal kinase 1
K^+^	Potassium
LC3B	Microtubule associated protein 1 light chain 3 beta
LCOS	Low cardiac output syndrome
LDL	Low-density lipoprotein
LFA-1	Lymphocyte function-associated antigen 1
LPS	Lipopolysaccharide
MAPK	Mitogen-associated protein kinase
MCP-1	Monocyte chemoattractant protein-1
MD-2	Myeloid differentiation-2
Mef2d	Myocyte enhancer factor 2d
Mg^2+^	Magnesium
MHC	Major histocompatibility complex
MIF	Macrophage migration inhibitory factor
MLCK	Myosin light chain kinase
MMP-1/9/13	Matrix metalloproteinase-1/9/13
mTOR	Mammalian target of rapamycin
MPTP	Mitochondrial permeability transition pore
MYC	Myelocytomatosis oncogene
MYH6	Myosin heavy chain 6
Na^+^	Sodium
NADPH	Nicotinamide adenine dinucleotide phosphate
NAFLD	Non-alcoholic fatty liver disease
NET	Neutrophil extracellular traps
NF-κB	Nuclear factor kappa B subunit 1
NK cells	Natural killer cells
NLR	Nod-like receptors
NLRP3	NLR family pyrin domain containing 3
NO	Nitric oxide
NOS	Nitric oxide synthase
NOX	Nicotinamide adenine dinucleotide phosphate (NADPH) Oxidase
p53	Tumor protein p53
PAF	Platelet activating factor
PAI-1	Plasminogen activator inhibitor 1
PAMP	Pathogen-associated molecular structures
PAR	Protease-activated receptors
PD-1	Programmed cell death protein 1
PDK1	Pyruvate dehydrogenase kinase 1
PEPCK	Phosphoenolpyruvate carboxykinase
PFK-2	Phosphofructokinase-2
PI3K	Phosphoinositide-3-kinase
PKC/Cδ/ε	Protein kinase C/C delta/C epsilon
PKM2	Pyruvate kinase M2
POAF	Postoperative atrial fibrillation
*PPARG*	*Peroxisome proliferator activated receptor gamma* (gene)
PPARγ	Peroxisome proliferator activated receptor gamma (protein)
PSGL-1	P-Selectin glycoprotein ligand-1
PTCA	Platelet-T-lymphocyte aggregates
PTDM	Post-transplantation diabetes mellitus
RAAS	Renin angiotensin system
RAGE	Receptor for advanced glycation end-products
RBC	Red blood cell
RNAi	Ribonucleic acid (RNA) interference
ROR-γt	Retinoic acid receptor-related orphan receptor gamma-t
ROS	Reactive oxygen species
RYR2	Ryanodine receptor 2
SERCA2a	ATPase sarcoplasmic/endoplasmic reticulum Ca2+ transporting 2
SGK1	Serum-glucocorticoid regulated kinase 1
SGLT2	Na^+^-Glucose cotransporter 2
sIL-6R	Soluble IL-6 receptor
SIRS	Systemic inflammatory response syndrome
SIRT1	Sirtuin 1
STAT1/3/5/6	Signal transducer and activator of transcription 1/3/5/6
T1DM	Type 1 diabetes mellitus
T2DM	Type 2 diabetes mellitus
T3DM	Type 3c diabetes mellitus
TAC	Transverse aortic constriction
TAG	Triacylglycerol
TCA	Tricarboxylic acid cycle
TCR	T-cell receptor
TGF-β	Transforming growth factor beta
TIMP-1/2	Tissue inhibitor of metalloproteinase-1/2
TLR/2/4	Toll-like receptor/-2/4
TNF-α	Tumor necrosis factor alpha
tPA	Tissue plasminogen activator
TRALI	Transfusion-related acute lung injury
UPR	Unfolded protein response
VCAM-1	Vascular cell adhesion molecule 1
VEGF	Vascular endothelial growth factor
VEGFA	Vascular endothelial growth factor A
VSMC	Vascular smooth muscle cells
WHO	World Health Organization

## References

[1] Diabetes. https://www.who.int/news-room/fact-sheets/detail/diabetes.

[2] Siam N.H., Snigdha N.N., Tabasumma N., Parvin I. (2024). Diabetes Mellitus and Cardiovascular Disease: Exploring Epidemiology, Pathophysiology, and Treatment Strategies. Rev. Cardiovasc. Med..

[3] Hattar L., Mumtaz T., El Mouhayyar C., Matevossian A., Johnstone M., Johnstone M., Veves A. (2023). The Effects and Treatment of Inflammation on Diabetes Mellitus and Cardiovascular Disease. Diabetes and Cardiovascular Disease.

[4] Squiccimarro E., Stasi A., Lorusso R., Paparella D. (2022). Narrative Review of the Systemic Inflammatory Reaction to Cardiac Surgery and Cardiopulmonary Bypass. Artif. Organs.

[5] Knapik P., Nadziakiewicz P., Urbanska E., Saucha W., Herdynska M., Zembala M. (2009). Cardiopulmonary Bypass Increases Postoperative Glycemia and Insulin Consumption After Coronary Surgery. Ann. Thorac. Surg..

[6] Antar S.A., Ashour N.A., Sharaky M., Khattab M., Ashour N.A., Zaid R.T., Roh E.J., Elkamhawy A., Al-Karmalawy A.A. (2023). Diabetes Mellitus: Classification, Mediators, and Complications; A Gate to Identify Potential Targets for the Development of New Effective Treatments. Biomed. Pharmacother..

[7] Martagon A.J., Zubirán R., González-Arellanes R., Praget-Bracamontes S., Rivera-Alcántara J.A., Aguilar-Salinas C.A. (2024). HDL Abnormalities in Type 2 Diabetes: Clinical Implications. Atherosclerosis.

[8] Yamada T., Kimura-Koyanagi M., Sakaguchi K., Ogawa W., Tamori Y. (2023). Obesity and Risk for Its Comorbidities Diabetes, Hypertension, and Dyslipidemia in Japanese Individuals Aged 65 Years. Sci. Rep..

[9] Mitrovic B., Gluvic Z.M., Obradovic M., Radunovic M., Rizzo M., Banach M., Isenovic E.R. (2022). Non-Alcoholic Fatty Liver Disease, Metabolic Syndrome, and Type 2 Diabetes Mellitus: Where Do We Stand Today?. Arch. Med. Sci..

[10] Sprenger L., Maechler M., Vonbank A., Larcher B., Mader A., Plattner T., Leiherer A., Muendlein A., Drexel H., Saely C.H. (2023). Type 2 Diabetes, Chronic Kidney Disease and Major Cardiovascular Events in Patients with Established Cardiovascular Disease. JACC.

[11] Borén J., Öörni K., Catapano A.L. (2024). The Link between Diabetes and Cardiovascular Disease. Atherosclerosis.

[12] Popoviciu M.S., Kaka N., Sethi Y., Patel N., Chopra H., Cavalu S. (2023). Type 1 Diabetes Mellitus and Autoimmune Diseases: A Critical Review of the Association and the Application of Personalized Medicine. J. Pers. Med..

[13] Vergès B. (2024). Cardiovascular Disease in Type 1 Diabetes, an Underestimated Danger: Epidemiological and Pathophysiological Data. Atherosclerosis.

[14] Rawshani A., Sattar N., Franzén S., Rawshani A., Hattersley A.T., Svensson A.-M., Eliasson B., Gudbjörnsdottir S. (2018). Excess Mortality and Cardiovascular Disease in Young Adults with Type 1 Diabetes in Relation to Age at Onset: A Nationwide, Register-Based Cohort Study. Lancet.

[15] Gottumukkala R.V.S.R.K., Lv H., Cornivelli L., Wagers A.J., Kwong R.Y., Bronson R., Stewart G.C., Schulze P.C., Chutkow W., Wolpert H.A. (2012). Myocardial Infarction Triggers Chronic Cardiac Autoimmunity in Type 1 Diabetes. Sci. Transl. Med..

[16] Dong B., Qi D., Yang L., Huang Y., Xiao X., Tai N., Wen L., Wong F.S. (2012). TLR4 Regulates Cardiac Lipid Accumulation and Diabetic Heart Disease in the Nonobese Diabetic Mouse Model of Type 1 Diabetes. Am. J. Physiol.-Heart Circ. Physiol..

[17] Tannus L.R.M., Drummond K.R.G., Clemente E.L.d.S., da Matta M.d.F.B., Gomes M.B., on behalf of the Brazilian Type 1 Diabetes Study Group (BrazDiab1SG) (2014). Predictors of Cardiovascular Autonomic Neuropathy in Patients with Type 1 Diabetes. Front. Endocrinol..

[18] de Ferranti S.D., de Boer I.H., Fonseca V., Fox C.S., Golden S.H., Lavie C.J., Magge S.N., Marx N., McGuire D.K., Orchard T.J. (2014). Type 1 Diabetes Mellitus and Cardiovascular Disease. Circulation.

[19] Ferrannini G., Manca M.L., Magnoni M., Andreotti F., Andreini D., Latini R., Maseri A., Maggioni A.P., Ostroff R.M., Williams S.A. (2020). Coronary Artery Disease and Type 2 Diabetes: A Proteomic Study. Diabetes Care.

[20] Swiatkiewicz I., Patel N.T., Villarreal-Gonzalez M., Taub P.R. (2024). Prevalence of Diabetic Cardiomyopathy in Patients with Type 2 Diabetes in a Large Academic Medical Center. BMC Med..

[21] Ma C.-X., Ma X.-N., Guan C.-H., Li Y.-D., Mauricio D., Fu S.-B. (2022). Cardiovascular Disease in Type 2 Diabetes Mellitus: Progress toward Personalized Management. Cardiovasc. Diabetol..

[22] Kautzky-Willer A., Leutner M., Harreiter J. (2023). Sex Differences in Type 2 Diabetes. Diabetologia.

[23] Yoo D., Kang M., Jung J. (2023). Risk of Ischemic Heart Disease in Patients With Postpancreatectomy Diabetes and Pancreatic Cancer: A Population-Based Study. J. Am. Heart Assoc..

[24] Wayne C.D., Benbetka C., Besner G.E., Narayanan S. (2024). Challenges of Managing Type 3c Diabetes in the Context of Pancreatic Resection, Cancer and Trauma. J. Clin. Med..

[25] Hjelmesæth J., Hartmann A., Leivestad T., Holdaas H., Sagedal S., Olstad M., Jenssen T. (2006). The Impact of Early-Diagnosed New-Onset Post-Transplantation Diabetes Mellitus on Survival and Major Cardiac Events. Kidney Int..

[26] Rysz J., Franczyk B., Radek M., Ciałkowska-Rysz A., Gluba-Brzózka A. (2021). Diabetes and Cardiovascular Risk in Renal Transplant Patients. Int. J. Mol. Sci..

[27] Meier-Kriesche H.-U., Schold J.D., Srinivas T.R., Reed A., Kaplan B. (2004). Kidney Transplantation Halts Cardiovascular Disease Progression in Patients with End-Stage Renal Diseas. Am. J. Transplant..

[28] Wu H.-X., Chu T.-Y., Iqbal J., Jiang H.-L., Li L., Wu Y.-X., Zhou H.-D. (2023). Cardio-Cerebrovascular Outcomes in MODY, Type 1 Diabetes, and Type 2 Diabetes: A Prospective Cohort Study. J. Clin. Endocrinol. Metab..

[29] Mao Y., Hu W., Xia B., Liu L., Han X., Liu Q. (2022). Association Between Gestational Diabetes Mellitus and the Risks of Type-Specific Cardiovascular Diseases. Front. Public Health.

[30] Chen A., Tan B., Du R., Chong Y.S., Zhang C., Koh A.S., Li L.-J. (2024). Gestational Diabetes Mellitus and Development of Intergenerational Overall and Subtypes of Cardiovascular Diseases: A Systematic Review and Meta-Analysis. Cardiovasc. Diabetol..

[31] Odegaard J.I., Chawla A. (2012). Connecting Type 1 and Type 2 Diabetes through Innate Immunity. Cold Spring Harb. Perspect. Med..

[32] Toyoshima Y., Nakamura K., Taguchi Y., Tokita R., Takeuchi S., Osawa H., Teramoto N., Sugihara H., Yoshizawa F., Yamanouchi K. (2025). Deletion of IRS-1 Leads to Growth Failure and Insulin Resistance with Downregulation of Liver and Muscle Insulin Signaling in Rats. Sci. Rep..

[33] Jaldin-Fincati J.R., Pavarotti M., Frendo-Cumbo S., Bilan P.J., Klip A. (2017). Update on GLUT4 Vesicle Traffic: A Cornerstone of Insulin Action. Trends Endocrinol. Metab..

[34] Cao J., Yu Y., Zhang Z., Chen X., Hu Z., Tong Q., Chang J., Feng X.-H., Lin X. (2018). SCP4 Promotes Gluconeogenesis Through FoxO1/3a Dephosphorylation. Diabetes.

[35] Markuns J.F., Wojtaszewski J.F.P., Goodyear L.J. (1999). Insulin and Exercise Decrease Glycogen Synthase Kinase-3 Activity by Different Mechanisms in Rat Skeletal Muscle. J. Biol. Chem..

[36] Takano A., Usui I., Haruta T., Kawahara J., Uno T., Iwata M., Kobayashi M. (2001). Mammalian Target of Rapamycin Pathway Regulates Insulin Signaling via Subcellular Redistribution of Insulin Receptor Substrate 1 and Integrates Nutritional Signals and Metabolic Signals of Insulin. Mol. Cell Biol..

[37] Lu X., Xie Q., Pan X., Zhang R., Zhang X., Peng G., Zhang Y., Shen S., Tong N. (2024). Type 2 Diabetes Mellitus in Adults: Pathogenesis, Prevention and Therapy. Signal Transduct. Target. Ther..

[38] Brown A.E., Walker M. (2016). Genetics of Insulin Resistance and the Metabolic Syndrome. Curr. Cardiol. Rep..

[39] Małodobra-Mazur M., Cierzniak A., Myszczyszyn A., Kaliszewski K., Dobosz T. (2021). Histone Modifications Influence the Insulin-Signaling Genes and Are Related to Insulin Resistance in Human Adipocytes. Int. J. Biochem. Cell Biol..

[40] Zhang J., Huang Y., Li H., Xu P., Liu Q., Sun Y., Zhang Z., Wu T., Tang Q., Jia Q. (2024). B3galt5 Functions as a PXR Target Gene and Regulates Obesity and Insulin Resistance by Maintaining Intestinal Integrity. Nat. Commun..

[41] Burhans M.S., Hagman D.K., Kuzma J.N., Schmidt K.A., Kratz M. (2019). Contribution of Adipose Tissue Inflammation to the Development of Type 2 Diabetes Mellitus. Compr. Physiol..

[42] Chandrasekaran P., Weiskirchen R. (2024). The Role of Obesity in Type 2 Diabetes Mellitus—An Overview. Int. J. Mol. Sci..

[43] Olefsky J.M., Glass C.K. (2010). Macrophages, Inflammation, and Insulin Resistance. Annu. Rev. Physiol..

[44] Yamauchi T., Nio Y., Maki T., Kobayashi M., Takazawa T., Iwabu M., Okada-Iwabu M., Kawamoto S., Kubota N., Kubota T. (2007). Targeted Disruption of AdipoR1 and AdipoR2 Causes Abrogation of Adiponectin Binding and Metabolic Actions. Nat. Med..

[45] Suzuki T., Gao J., Ishigaki Y., Kondo K., Sawada S., Izumi T., Uno K., Kaneko K., Tsukita S., Takahashi K. (2017). ER Stress Protein CHOP Mediates Insulin Resistance by Modulating Adipose Tissue Macrophage Polarity. Cell Rep..

[46] Kang K., Reilly S.M., Karabacak V., Gangl M.R., Fitzgerald K., Hatano B., Lee C.-H. (2008). Adipocyte-Derived Th2 Cytokines and Myeloid PPARδ Regulate Macrophage Polarization and Insulin Sensitivity. Cell Metab..

[47] Li X., Xiao G.-Y., Guo T., Song Y.-J., Li Q.-M. (2022). Potential Therapeutic Role of Pyroptosis Mediated by the NLRP3 Inflammasome in Type 2 Diabetes and Its Complications. Front. Endocrinol..

[48] Cucak H., Grunnet L.G., Rosendahl A. (2014). Accumulation of M1-like Macrophages in Type 2 Diabetic Islets Is Followed by a Systemic Shift in Macrophage Polarization. J. Leukoc. Biol..

[49] León-Pedroza J.I., González-Tapia L.A., del Olmo-Gil E., Castellanos-Rodríguez D., Escobedo G., González-Chávez A. (2015). Low-Grade Systemic Inflammation and the Development of Metabolic Diseases: From the Molecular Evidence to the Clinical Practice. Cirugía Y Cir..

[50] Kunz H.E., Hart C.R., Gries K.J., Parvizi M., Laurenti M., Man C.D., Moore N., Zhang X., Ryan Z., Polley E.C. (2021). Adipose Tissue Macrophage Populations and Inflammation Are Associated with Systemic Inflammation and Insulin Resistance in Obesity. Am. J. Physiol.-Endocrinol. Metab..

[51] Shirakawa K., Yan X., Shinmura K., Endo J., Kataoka M., Katsumata Y., Yamamoto T., Anzai A., Isobe S., Yoshida N. (2016). Obesity Accelerates T Cell Senescence in Murine Visceral Adipose Tissue. J. Clin. Investig..

[52] McLaughlin T., Liu L.-F., Lamendola C., Shen L., Morton J., Rivas H., Winer D., Tolentino L., Choi O., Zhang H. (2014). T-Cell Profile in Adipose Tissue Is Associated With Insulin Resistance and Systemic Inflammation in Humans. Arterioscler. Thromb. Vasc. Biol..

[53] Rocha V.Z., Folco E.J., Sukhova G., Shimizu K., Gotsman I., Vernon A.H., Libby P. (2008). Interferon-Gamma, a Th1 Cytokine, Regulates Fat Inflammation: A Role for Adaptive Immunity in Obesity. Circ. Res..

[54] Winer S., Chan Y., Paltser G., Truong D., Tsui H., Bahrami J., Dorfman R., Wang Y., Zielenski J., Mastronardi F. (2009). Normalization of Obesity-Associated Insulin Resistance through Immunotherapy. Nat. Med..

[55] Chen X., Shi C., He M., Xiong S., Xia X. (2023). Endoplasmic Reticulum Stress: Molecular Mechanism and Therapeutic Targets. Signal Transduct. Target. Ther..

[56] Thakkar H., Vincent V., Chaurasia B. (2025). Ceramide Signaling in Immunity: A Molecular Perspective. Lipids Health Dis..

[57] Delcheva G., Stefanova K., Stankova T. (2024). Ceramides—Emerging Biomarkers of Lipotoxicity in Obesity, Diabetes, Cardiovascular Diseases, and Inflammation. Diseases.

[58] Wang X., Zhu L., Liu J., Ma Y., Qiu C., Liu C., Gong Y., Yuwen Y., Guan G., Zhang Y. (2024). Palmitic Acid in Type 2 Diabetes Mellitus Promotes Atherosclerotic Plaque Vulnerability via Macrophage Dll4 Signaling. Nat. Commun..

[59] Ohashi K., Parker J.L., Ouchi N., Higuchi A., Vita J.A., Gokce N., Pedersen A.A., Kalthoff C., Tullin S., Sams A. (2010). Adiponectin Promotes Macrophage Polarization toward an Anti-Inflammatory Phenotype. J. Biol. Chem..

[60] Xia C., Rao X., Zhong J. (2017). Role of T Lymphocytes in Type 2 Diabetes and Diabetes-Associated Inflammation. J. Diabetes Res..

[61] Meshkani R., Vakili S. (2016). Tissue Resident Macrophages: Key Players in the Pathogenesis of Type 2 Diabetes and Its Complications. Clin. Chim. Acta.

[62] Pavlou S., Lindsay J., Ingram R., Xu H., Chen M. (2018). Sustained High Glucose Exposure Sensitizes Macrophage Responses to Cytokine Stimuli but Reduces Their Phagocytic Activity. BMC Immunol..

[63] Witcoski Junior L., de Lima J.D., Somensi A.G., de Souza Santos L.B., Paschoal G.L., Uada T.S., Bastos T.S.B., de Paula A.G.P., Dos Santos Luz R.B., Czaikovski A.P. (2024). Metabolic Reprogramming of Macrophages in the Context of Type 2 Diabetes. Eur. J. Med. Res..

[64] Vlassara H., Uribarri J. (2013). Advanced Glycation End Products (AGE) and Diabetes: Cause, Effect, or Both?. Curr. Diabetes Rep..

[65] Lee J., Yun J.-S., Ko S.-H. (2022). Advanced Glycation End Products and Their Effect on Vascular Complications in Type 2 Diabetes Mellitus. Nutrients.

[66] Petrie J.R., Guzik T.J., Touyz R.M. (2018). Diabetes, Hypertension, and Cardiovascular Disease: Clinical Insights and Vascular Mechanisms. Can. J. Cardiol..

[67] Rajbhandari J., Fernandez C.J., Agarwal M., Yeap B.X.Y., Pappachan J.M. (2021). Diabetic Heart Disease: A Clinical Update. World J. Diabetes.

[68] Lei X., Qiu S., Yang G., Wu Q. (2023). Adiponectin and Metabolic Cardiovascular Diseases: Therapeutic Opportunities and Challenges. Genes. Dis..

[69] Avagimyan A., Fogacci F., Pogosova N., Kakrurskiy L., Kogan E., Urazova O., Kobalava Z., Mikhaleva L., Vandysheva R., Zarina G. (2024). Diabetic Cardiomyopathy: 2023 Update by the International Multidisciplinary Board of Experts. Curr. Probl. Cardiol..

[70] Ussher J.R. (2014). The Role of Cardiac Lipotoxicity in the Pathogenesis of Diabetic Cardiomyopathy. Expert. Rev. Cardiovasc. Ther..

[71] Guo W., Yang C., Zou J., Yu T., Li M., He R., Chen K., Hell R.C.R., Gross E.R., Zou X. (2024). Interleukin-1β Polarization in M1 Macrophage Mediates Myocardial Fibrosis in Diabetes. Int. Immunopharmacol..

[72] Zi C., He L., Yao H., Ren Y., He T., Gao Y. (2022). Changes of Th17 Cells, Regulatory T Cells, Treg/Th17, IL-17 and IL-10 in Patients with Type 2 Diabetes Mellitus: A Systematic Review and Meta-Analysis. Endocrine.

[73] Nekoua M.P., Fachinan R., Atchamou A.K., Nouatin O., Amoussou-Guenou D., Amoussou-Guenou M.K., Moutairou K., Yessoufou A. (2016). Modulation of Immune Cells and Th1/Th2 Cytokines in Insulin-Treated Type 2 Diabetes Mellitus. Afr. Health Sci..

[74] Olson N.C., Doyle M.F., de Boer I.H., Huber S.A., Jenny N.S., Kronmal R.A., Psaty B.M., Tracy R.P. (2015). Associations of Circulating Lymphocyte Subpopulations with Type 2 Diabetes: Cross-Sectional Results from the Multi-Ethnic Study of Atherosclerosis (MESA). PLoS ONE.

[75] Cheng Y., Wang Y., Yin R., Xu Y., Zhang L., Zhang Y., Yang L., Zhao D. (2023). Central Role of Cardiac Fibroblasts in Myocardial Fibrosis of Diabetic Cardiomyopathy. Front. Endocrinol..

[76] Zhang K., Li Y., Ge X., Meng L., Kong J., Meng X. (2024). Regulatory T Cells Protect against Diabetic Cardiomyopathy in Db/Db Mice. J. Diabetes Investig..

[77] Zhang X., Ma X., Zhao M., Zhang B., Chi J., Liu W., Chen W., Fu Y., Liu Y., Yin X. (2015). H2 and H3 Relaxin Inhibit High Glucose-Induced Apoptosis in Neonatal Rat Ventricular Myocytes. Biochimie.

[78] Yang M., Zheng J., Miao Y., Wang Y., Cui W., Guo J., Qiu S., Han Y., Jia L., Li H. (2012). Serum-Glucocorticoid Regulated Kinase 1 Regulates Alternatively Activated Macrophage Polarization Contributing to Angiotensin II–Induced Inflammation and Cardiac Fibrosis. Arterioscler. Thromb. Vasc. Biol..

[79] Li J.-M., Gall N.P., Grieve D.J., Chen M., Shah A.M. (2002). Activation of NADPH Oxidase during Progression of Cardiac Hypertrophy to Failure. Hypertension.

[80] Jia G., DeMarco V.G., Sowers J.R. (2016). Insulin Resistance and Hyperinsulinaemia in Diabetic Cardiomyopathy. Nat. Rev. Endocrinol..

[81] Zlobine I., Gopal K., Ussher J.R. (2016). Lipotoxicity in Obesity and Diabetes-Related Cardiac Dysfunction. Biochim. Biophys. Acta.

[82] Luo S., Xu R., Xie P., Liu X., Ling C., Liu Y., Zhang X., Xia Z., Chen Z., Tang J. (2024). EGFR of Platelet Regulates Macrophage Activation and Bacterial Phagocytosis Function. J. Inflamm..

[83] Ferreira I.A., Eybrechts K.L., Mocking A.I.M., Kroner C., Akkerman J.-W.N. (2004). IRS-1 Mediates Inhibition of Ca2+ Mobilization by Insulin via the Inhibitory G-Protein Gi. J. Biol. Chem..

[84] Lee W.J., Tateya S., Cheng A.M., Rizzo-DeLeon N., Wang N.F., Handa P., Wilson C.L., Clowes A.W., Sweet I.R., Bomsztyk K. (2015). M2 Macrophage Polarization Mediates Anti-Inflammatory Effects of Endothelial Nitric Oxide Signaling. Diabetes.

[85] Guzik T.J., Mussa S., Gastaldi D., Sadowski J., Ratnatunga C., Pillai R., Channon K.M. (2002). Mechanisms of Increased Vascular Superoxide Production in Human Diabetes Mellitus: Role of NAD(P)H Oxidase and Endothelial Nitric Oxide Synthase. Circulation.

[86] Lemkes B.A., Hermanides J., Devries J.H., Holleman F., Meijers J.C.M., Hoekstra J.B.L. (2010). Hyperglycemia: A Prothrombotic Factor?. J. Thromb. Haemost..

[87] Xia Y., Gao D., Wang X., Liu B., Shan X., Sun Y., Ma D. (2024). Role of Treg Cell Subsets in Cardiovascular Disease Pathogenesis and Potential Therapeutic Targets. Front. Immunol..

[88] Glöckner A., Ossmann S., Ginther A., Kang J., Borger M.A., Hoyer A., Dieterlen M.-T. (2021). Relevance and Recommendations for the Application of Cardioplegic Solutions in Cardiopulmonary Bypass Surgery in Pigs. Biomedicines.

[89] Nielsen E.W., Miller Y., Brekke O.-L., Grond J., Duong A.H., Fure H., Ludviksen J.K., Pettersen K., Reubsaet L., Solberg R. (2022). A Novel Porcine Model of Ischemia-Reperfusion Injury After Cross-Clamping the Thoracic Aorta Revealed Substantial Cardiopulmonary, Thromboinflammatory and Biochemical Changes Without Effect of C1-Inhibitor Treatment. Front. Immunol..

[90] Pagehgiri H.D., Puruhito I., Aditiawarman A. (2023). Ischemia Reperfusion Injury on Temporary Aortic Cross-Clamping. Ital. J. Vasc. Endovasc. Surg..

[91] Ergene S., Hemsinli D., Karakisi S.O., Tümkaya L., Mercantepe T., Yilmaz A., Yel I. (2023). Resveratrol Attenuates Degeneration and Apoptosis of Cardiomyocytes Induced by Aortic Clamping. Braz. J. Cardiovasc. Surg..

[92] Prata M.P., Jaldin R.G., Lourenção P.L.T.d.A., Sobreira M.L., Yoshida R.d.A., Terra S.A., Viero R.M., Yoshida W.B. (2020). Acute Aortic Wall Injury Caused by Aortic Cross-Clamping: Morphological and Biomechanical Study of the Aorta in a Swine Model of Three Aortic Surgery Approaches. J. Vasc. Bras..

[93] Rylski B., Beyersdorf F., Czerny M., Siepe M. (2016). Two Aortic Ruptures in Two Months—Role of Cross–Clamp-Associated Late Injury. Ann. Vasc. Surg..

[94] Nguyen Q.-S., Banks D., Awad A.S., DiNardo J., Huang J., Hancock M.F. (2025). Strategies of Myocardial Protection During Cardiopulmonary Bypass. Clinical Perfusion for Cardiac Surgery: A Step-by-Step Guide to the Fundamentals.

[95] Aykut G., Ulugöl H., Aksu U., Akin S., Karabulut H., Alhan C., Toraman F., Ince C. (2022). Microcirculatory Response to Blood vs. Crystalloid Cardioplegia During Coronary Artery Bypass Grafting With Cardiopulmonary Bypass. Front. Med..

[96] Brown A.J., Chambers D.J. (2021). Physiology and Cardioplegia: Safety in Operating. Surgery.

[97] Francica A., Tonelli F., Rossetti C., Tropea I., Luciani G.B., Faggian G., Dobson G.P., Onorati F. (2021). Cardioplegia between Evolution and Revolution: From Depolarized to Polarized Cardiac Arrest in Adult Cardiac Surgery. J. Clin. Med..

[98] Jonjev Ź.S., Schwertz D.W., Beck J.M., Ross J.D., Law W.R. (2003). Subcellular Distribution of Protein Kinase C Isozymes during Cardioplegic Arrest. J. Thorac. Cardiovasc. Surg..

[99] Sodha N.R., Clements R.T., Bianchi C., Sellke F.W. (2008). Cardiopulmonary Bypass with Cardioplegic Arrest Activates Protein Kinase C in the Human Myocardium. J. Am. Coll. Surg..

[100] Khoynezhad A., Jalali Z., Tortolani A.J. (2004). Apoptosis: Pathophysiology and Therapeutic Implications for the Cardiac Surgeon. Ann. Thorac. Surg..

[101] Fischer U.M., Cox C.S., Laine G.A., Mehlhorn U., Bloch W., Allen S.J. (2007). Induction of Cardioplegic Arrest Immediately Activates the Myocardial Apoptosis Signal Pathway. Am. J. Physiol.-Heart Circ. Physiol..

[102] Shuja F., Tabbara M., Li Y., Liu B., Butt M.U., Velmahos G.C., DeMoya M., Alam H.B. (2009). Profound Hypothermia Decreases Cardiac Apoptosis through Akt Survival Pathway. J. Am. Coll. Surg..

[103] Ji M.J., Hong J.H. (2021). A Cardioplegic Solution with an Understanding of a Cardiochannelopathy. Antioxidants.

[104] Yeh C.-H., Wang Y.-C., Wu Y.-C., Chu J.-J., Lin P.J. (2003). Continuous Tepid Blood Cardioplegia Can Preserve Coronary Endothelium and Ameliorate the Occurrence of Cardiomyocyte Apoptosis. Chest.

[105] Panconesi R., Widmer J., Carvalho M.F., Eden J., Dondossola D., Dutkowski P., Schlegel A. (2022). Mitochondria and Ischemia Reperfusion Injury. Curr. Opin. Organ Transplant..

[106] De Hert S., Moerman A. (2015). Myocardial Injury and Protection Related to Cardiopulmonary Bypass. Best Pract. Res. Clin. Anaesthesiol..

[107] Muraki S., Morris C.D., Budde J.M., Zhao Z.-Q., Guyton R.A., Vinten-Johansen J. (2003). Blood Cardioplegia Supplementation with the Sodium-Hydrogen Ion Exchange Inhibitor Cariporide to Attenuate Infarct Size and Coronary Artery Endothelial Dysfunction after Severe Regional Ischemia in a Canine Model. J. Thorac. Cardiovasc. Surg..

[108] de Oliveira M.A.B., Brandi A.C., dos Santos C.A., Botelho P.H.H., Cortez J.L.L., Goissis G., Braile D.M. (2014). The Calcium Paradox—What Should We Have to Fear?. Rev. Bras. Cir. Cardiovasc..

[109] Saldanha C., Hearse D.J. (1994). Cardioplegia and Vascular Injury: Dissociation of the Effects of Ischemia from Those of the Cardioplegic Solution. J. Thorac. Cardiovasc. Surg..

[110] Keller M.W., Geddes L., Spotnitz W., Kaul S., Duling B.R. (1991). Microcirculatory Dysfunction Following Perfusion with Hyperkalemic, Hypothermic, Cardioplegic Solutions and Blood Reperfusion. Effects of Adenosine. Circulation.

[111] Xue H.-M., Hou H.-T., Sun W.-T., Wang S.-F., Guo S., Yang Q., He G.-W. (2022). Del Nido Cardioplegia Better Preserves Cardiac Diastolic Function but Histidine–Tryptophan–Ketoglutarate Is Better for Endothelial Function. Eur. J. Cardiothorac. Surg..

[112] Ferrera R., Michel P., Ovize M. (2005). Paradoxical Toxicity of Cardioplegic Compounds on Ischemic Cardiomyocyte Using Optimal Design Strategy. J. Heart Lung Transplant..

[113] Shimoda T., Liu C., Mathis B.J., Goto Y., Ageyama N., Kato H., Matsubara M., Ohigashi T., Gosho M., Suzuki Y. (2023). Effect of Cardiopulmonary Bypass on Coagulation Factors II, VII and X in a Primate Model: An Exploratory Pilot Study. Interdiscip. Cardiovasc. Thorac. Surg..

[114] Volk L.E., Mavroudis C.D., Ko T., Hallowell T., Delso N., Roberts A.L., Starr J., Landis W., Lin Y., Hefti M. (2021). Increased Cerebral Mitochondrial Dysfunction and Reactive Oxygen Species with Cardiopulmonary Bypass. Eur. J. Cardiothorac. Surg..

[115] Kramer R.S., Herron C.R., Groom R.C., Brown J.R. (2015). Acute Kidney Injury Subsequent to Cardiac Surgery. J. Extra Corpor. Technol..

[116] Woods B.D., Sladen R.N. (2009). Perioperative Considerations for the Patient with Asthma and Bronchospasm. Br. J. Anaesth..

[117] Kim L., Nguyen H.-Y., Senawong T., Wei C. (2025). Protamine-Related Non-Cardiogenic Pulmonary Edema during Routine Heparin Reversal for Cardiopulmonary Bypass. Med. Rep..

[118] Salomon J.D., Qiu H., Feng D., Owens J., Khailova L., Osorio Lujan S., Iguidbashian J., Chhonker Y.S., Murry D.J., Riethoven J.-J. (2023). Piglet Cardiopulmonary Bypass Induces Intestinal Dysbiosis and Barrier Dysfunction Associated with Systemic Inflammation. Dis. Model. Mech..

[119] Rhaleb N.-E., Yang X.-P., Carretero O.A. (2011). The Kallikrein-Kinin System as a Regulator of Cardiovascular and Renal Function. Compr. Physiol..

[120] Chatterjee K., Thornton J.L., Bauer J.W., Vogler E.A., Siedlecki C.A. (2009). Moderation of Prekallikrein-Factor XII Interactions in Surface Activation of Coagulation by Protein-Adsorption Competition. Biomaterials.

[121] Bekassy Z., Lopatko Fagerström I., Bader M., Karpman D. (2022). Crosstalk between the Renin–Angiotensin, Complement and Kallikrein–Kinin Systems in Inflammation. Nat. Rev. Immunol..

[122] Banerjee D., Feng J., Sellke F.W. (2024). Strategies to Attenuate Maladaptive Inflammatory Response Associated with Cardiopulmonary Bypass. Front. Surg..

[123] Somer F.D., Belleghem Y.V., Caes F., François K., Overbeke H.V., Arnout J., Taeymans Y., Nooten G.V. (2002). Tissue Factor as the Main Activator of the Coagulation System during Cardiopulmonary Bypass. J. Thorac. Cardiovasc. Surg..

[124] Witkowski M., Landmesser U., Rauch U. (2016). Tissue Factor as a Link between Inflammation and Coagulation. Trends Cardiovasc. Med..

[125] Sang Y., Roest M., de Laat B., de Groot P.G., Huskens D. (2021). Interplay between Platelets and Coagulation. Blood Rev..

[126] Kelchtermans H., Pelkmans L., Bouwhuis A., Schurgers E., Lindhout T., Huskens D., Miszta A., Hemker H.C., Lancé M.D., de Laat B. (2016). Simultaneous Measurement of Thrombin Generation and Fibrin Formation in Whole Blood under Flow Conditions. Thromb. Haemost..

[127] Parolari A., Colli S., Mussoni L., Eligini S., Naliato M., Wang X., Gandini S., Tremoli E., Biglioli P., Alamanni F. (2003). Coagulation and Fibrinolytic Markers in a Two-Month Follow-up of Coronary Bypass Surgery. J. Thorac. Cardiovasc. Surg..

[128] Olson S.A., Osborn B.K., Cotton M.E., Krocker J.D., Koami H., White N., Cardenas J.C. (2024). Fibrinogen Fragment X Mediates Endothelial Barrier Disruption via Suppression of VE-Cadherin. J. Surg. Res..

[129] Amara U., Rittirsch D., Flierl M., Bruckner U., Klos A., Gebhard F., Lambris J.D., Huber-Lang M. (2008). Interaction Between the Coagulation and Complement System. Adv. Exp. Med. Biol..

[130] Kaur J., Woodman R.C., Ostrovsky L., Kubes P. (2001). Selective Recruitment of Neutrophils and Lymphocytes by Thrombin: A Role for NF-κB. Am. J. Physiol.-Heart Circ. Physiol..

[131] Gould T.J., Vu T.T., Swystun L.L., Dwivedi D.J., Mai S.H.C., Weitz J.I., Liaw P.C. (2014). Neutrophil Extracellular Traps Promote Thrombin Generation Through Platelet-Dependent and Platelet-Independent Mechanisms. Arterioscler. Thromb. Vasc. Biol..

[132] Beaubien-Souligny W., Neagoe P.-E., Gagnon D., Denault A.Y., Sirois M.G. (2019). Increased Circulating Levels of Neutrophil Extracellular Traps During Cardiopulmonary Bypass. CJC Open.

[133] Jung Y., Choi J.W., Hwang H.Y., Gu J.Y., Kim K.H., Kim H.K. (2024). Elevated Circulating Levels of Neutrophil Extracellular Traps after Cardiopulmonary Bypass Surgery as Risk Factors of Postoperative Atrial Fibrillation and Mortality. J. Thorac. Dis..

[134] Chenoweth D.E., Cooper S.W., Hugli T.E., Stewart R.W., Blackstone E.H., Kirklin J.W. (1981). Complement Activation during Cardiopulmonary Bypass: Evidence for Generation of C3a and C5a Anaphylatoxins. N. Engl. J. Med..

[135] Fitch J.C.K., Rollins S., Matis L., Alford B., Aranki S., Collard C.D., Dewar M., Elefteriades J., Hines R., Kopf G. (1999). Pharmacology and Biological Efficacy of a Recombinant, Humanized, Single-Chain Antibody C5 Complement Inhibitor in Patients Undergoing Coronary Artery Bypass Graft Surgery With Cardiopulmonary Bypass. Circulation.

[136] Kefalogianni R., Kamani F., Gaspar M., Aw T., Donovan J., Laffan M., Pickering M.C., Arachchillage D.J. (2022). Complement Activation during Cardiopulmonary Bypass and Association with Clinical Outcomes. eJHaem.

[137] Denk S., Taylor R.P., Wiegner R., Cook E.M., Lindorfer M.A., Pfeiffer K., Paschke S., Eiseler T., Weiss M., Barth E. (2017). Complement C5a-Induced Changes in Neutrophil Morphology during Inflammation. Scand. J. Immunol..

[138] Mannes M., Pechtl V., Hafner S., Dopler A., Eriksson O., Manivel V.A., Wohlgemuth L., Messerer D.A.C., Schrezenmeier H., Ekdahl K.N. (2023). Complement and Platelets: Prothrombotic Cell Activation Requires Membrane Attack Complex–Induced Release of Danger Signals. Blood Adv..

[139] Aydin N.B., Gercekoglu H., Aksu B., Ozkul V., Sener T., Kıygıl İ., Turkoglu T., Cimen S., Babacan F., Demirtas M. (2003). Endotoxemia in Coronary Artery Bypass Surgery: A Comparison of the off-Pump Technique and Conventional Cardiopulmonary Bypass. J. Thorac. Cardiovasc. Surg..

[140] Ciesielska A., Krawczyk M., Sas-Nowosielska H., Hromada-Judycka A., Kwiatkowska K. (2022). CD14 Recycling Modulates LPS-Induced Inflammatory Responses of Murine Macrophages. Traffic.

[141] Dayang E.-Z., Plantinga J., Ter Ellen B., van Meurs M., Molema G., Moser J. (2019). Identification of LPS-Activated Endothelial Subpopulations With Distinct Inflammatory Phenotypes and Regulatory Signaling Mechanisms. Front. Immunol..

[142] Siepe M., Goebel U., Mecklenburg A., Doenst T., Benk C., Stein P., Beyersdorf F., Loop T., Schlensak C. (2008). Pulsatile Pulmonary Perfusion During Cardiopulmonary Bypass Reduces the Pulmonary Inflammatory Response. Ann. Thorac. Surg..

[143] Luecht J., Pauli C., Seiler R., Herre A.-L., Brankova L., Berger F., Schmitt K.R.L., Tong G. (2024). Prolonged Extracorporeal Circulation Leads to Inflammation and Higher Expression of Mediators of Vascular Permeability Through Activation of STAT3 Signaling Pathway in Macrophages. Int. J. Mol. Sci..

[144] Dreyer W.J., Phillips S.C., Lindsey M.L., Jackson P., Bowles N.E., Michael L.H., Entman M.L. (2000). Interleukin 6 Induction in the Canine Myocardium after Cardiopulmonary Bypass. J. Thorac. Cardiovasc. Surg..

[145] Fontes J.A., Rose N.R., Čiháková D. (2015). The Varying Faces of IL-6: From Cardiac Protection to Cardiac Failure. Cytokine.

[146] Puchinger J., Ryz S., Nixdorf L., Edlinger-Stanger M., Lassnigg A., Wiedemann D., Hiesmayr M., Spittler A., Bernardi M.H. (2022). Characteristics of Interleukin-6 Signaling in Elective Cardiac Surgery—A Prospective Cohort Study. J. Clin. Med..

[147] Luo C., Xie X., Feng X., Lei B., Fang C., Li Y., Cai X., Ling G., Zheng B. (2020). Deficiency of Interleukin-36 Receptor Protected Cardiomyocytes from Ischemia-Reperfusion Injury in Cardiopulmonary Bypass. Med. Sci. Monit..

[148] Zhang W., Huang Q., Zeng Z., Wu J., Zhang Y., Chen Z. (2017). Sirt1 Inhibits Oxidative Stress in Vascular Endothelial Cells. Oxid. Med. Cell Longev..

[149] Lou X., Duan S., Li M., Yuan Y., Chen S., Wang Z., Wang Z., Sun L. (2023). IL-36α Inhibits Melanoma by Inducing pro-Inflammatory Polarization of Macrophages. Cancer Immunol. Immunother..

[150] Flier S., Concepcion A.N., Versteeg D., Kappen T.H., Hoefer I.E., de Lange D.W., Pasterkamp G., Buhre W.F. (2015). Monocyte Hyporesponsiveness and Toll-like Receptor Expression Profiles in Coronary Artery Bypass Grafting and Its Clinical Implications for Postoperative Inflammatory Response and Pneumonia: An Observational Cohort Study. Eur. J. Anaesthesiol..

[151] Gaudriot B., Uhel F., Gregoire M., Gacouin A., Biedermann S., Roisne A., Flecher E., Le Tulzo Y., Tarte K., Tadié J.-M. (2015). Immune Dysfunction After Cardiac Surgery with Cardiopulmonary Bypass: Beneficial Effects of Maintaining Mechanical Ventilation. Shock.

[152] Lesouhaitier M., Belicard F., Tadié J.-M. (2024). Cardiopulmonary Bypass and VA-ECMO Induced Immune Dysfunction: Common Features and Differences, a Narrative Review. Crit. Care.

[153] Hou L., Yang Z., Wang Z., Zhang X., Zhao Y., Yang H., Zheng B., Tian W., Wang S., He Z. (2018). NLRP3/ASC-Mediated Alveolar Macrophage Pyroptosis Enhances HMGB1 Secretion in Acute Lung Injury Induced by Cardiopulmonary Bypass. Lab. Investig..

[154] Kotani N., Hashimoto H., Sessler D.I., Muraoka M., Wang J.-S., O’Connor M.F., Matsuki A. (2000). Cardiopulmonary Bypass Produces Greater Pulmonary than Systemic Proinflammatory Cytokines. Anesth. Analg..

[155] Sumaiya K., Langford D., Natarajaseenivasan K., Shanmughapriya S. (2022). Macrophage Migration Inhibitory Factor (MIF): A Multifaceted Cytokine Regulated by Genetic and Physiological Strategies. Pharmacol. Ther..

[156] Toso C., Emamaullee J.A., Merani S., Shapiro A.M.J. (2008). The Role of Macrophage Migration Inhibitory Factor on Glucose Metabolism and Diabetes. Diabetologia.

[157] Bernhagen J., Krohn R., Lue H., Gregory J.L., Zernecke A., Koenen R.R., Dewor M., Georgiev I., Schober A., Leng L. (2007). MIF Is a Noncognate Ligand of CXC Chemokine Receptors in Inflammatory and Atherogenic Cell Recruitment. Nat. Med..

[158] Miller E.J., Li J., Leng L., McDonald C., Atsumi T., Bucala R., Young L.H. (2008). Macrophage Migration Inhibitory Factor Stimulates AMP-Activated Protein Kinase in the Ischaemic Heart. Nature.

[159] Atsumi T., Cho Y.-R., Leng L., McDonald C., Yu T., Danton C., Hong E.-G., Mitchell R.A., Metz C., Niwa H. (2007). The Proinflammatory Cytokine Macrophage Migration Inhibitory Factor Regulates Glucose Metabolism during Systemic Inflammation. J. Immunol..

[160] Stoppe C., Werker T., Rossaint R., Dollo F., Lue H., Wonisch W., Menon A., Goetzenich A., Bruells C.S., Coburn M. (2013). What Is the Significance of Perioperative Release of Macrophage Migration Inhibitory Factor in Cardiac Surgery?. Antioxid. Redox Signal..

[161] Stoppe C., Grieb G., Rossaint R., Simons D., Coburn M., Götzenich A., Strüssmann T., Pallua N., Bernhagen J., Rex S. (2012). High Postoperative Blood Levels of Macrophage Migration Inhibitory Factor Are Associated with Less Organ Dysfunction in Patients after Cardiac Surgery. Mol. Med..

[162] Furtado de Mendonça-Filho H.T., Gomes R.V., Campos L.A.d.A., Tura B., Nunes E.M., Gomes R., Bozza F., Bozza P.T., Castro-Faria-Neto H.C. (2004). Circulating Levels of Macrophage Migration Inhibitory Factor Are Associated with Mild Pulmonary Dysfunction After Cardiopulmonary Bypass. Shock.

[163] Gabrilovich D.I., Nagaraj S. (2009). Myeloid-Derived Suppressor Cells as Regulators of the Immune System. Nat. Rev. Immunol..

[164] Rodríguez-López J.M., Iglesias-González J.L., Lozano-Sánchez F.S., Palomero-Rodríguez M.Á., Sánchez-Conde P. (2022). Inflammatory Response, Immunosuppression and Arginase Activity after Cardiac Surgery Using Cardiopulmonary Bypass. J. Clin. Med..

[165] Li W.-J., Peng Y.-X., Zhao L.-Q., Wang H.-Y., Liu W., Bai K., Chen S., Lu Y., Huang J. (2024). T-Cell Lymphopenia Is Associated with an Increased Infecting Risk in Children after Cardiopulmonary Bypass. Pediatr. Res..

[166] Farhid F., Hosseini E., Kargar F., Ghasemzadeh M. (2025). Interplay between Platelet and T Lymphocyte after Coronary Artery Bypass Grafting (CABG): Evidence for Platelet Mediated Post-CABG Immunomodulation. Microvasc. Res..

[167] Hosseini E., Ahmadi J., Kargar F., Ghasemzadeh M. (2024). Coronary Artery Bypass Grafting (CABG) Induces pro-Inflammatory and Immunomodulatory Phenotype of Platelets in the Absence of a pro-Aggregatory State. Microvasc. Res..

[168] Zhou W., Yang Y., Feng Z., Zhang Y., Chen Y., Yu T., Wang H. (2024). Inhibition of Caspase-1-Dependent Pyroptosis Alleviates Myocardial Ischemia/Reperfusion Injury during Cardiopulmonary Bypass (CPB) in Type 2 Diabetic Rats. Sci. Rep..

[169] Schweizer T., Nossen C.M., Galova B., Schild C., Huber M., Bally L., Vogt A., Siepe M., Nagler M., Fischer K. (2025). In Vitro Investigation of Insulin Dynamics During 4 Hours of Simulated Cardiopulmonary Bypass. Anesth. Analg..

[170] Cao Y., Yang T., Yu S., Sun G., Gu C., Yi D. (2013). Relationships of Adiponectin with Markers of Systemic Inflammation and Insulin Resistance in Infants Undergoing Open Cardiac Surgery. Mediat. Inflamm..

[171] Kremen J., Dolinkova M., Krajickova J., Blaha J., Anderlova K., Lacinova Z., Haluzikova D., Bosanska L., Vokurka M., Svacina S. (2006). Increased Subcutaneous and Epicardial Adipose Tissue Production of Proinflammatory Cytokines in Cardiac Surgery Patients: Possible Role in Postoperative Insulin Resistance. J. Clin. Endocrinol. Metab..

[172] de Lange F., Dieleman J.M., Jungwirth B., Kalkman C.J. (2007). Effects of Cardiopulmonary Bypass on Neurocognitive Performance and Cytokine Release in Old and Diabetic Rats. Br. J. Anaesth..

[173] Hoedemaekers C.W., Pickkers P., Netea M.G., van Deuren M., Van der Hoeven J.G. (2005). Intensive Insulin Therapy Does Not Alter the Inflammatory Response in Patients Undergoing Coronary Artery Bypass Grafting: A Randomized Controlled Trial [ISRCTN95608630]. Crit. Care.

[174] Esposito K., Nappo F., Marfella R., Giugliano G., Giugliano F., Ciotola M., Quagliaro L., Ceriello A., Giugliano D. (2002). Inflammatory Cytokine Concentrations Are Acutely Increased by Hyperglycemia in Humans: Role of Oxidative Stress. Circulation.

[175] Wasmuth H.E., Kunz D., Graf J., Stanzel S., Purucker E.A., Koch A., Gartung C., Heintz B., Gressner A.M., Matern S. (2004). Hyperglycemia at Admission to the Intensive Care Unit Is Associated with Elevated Serum Concentrations of Interleukin-6 and Reduced Ex Vivo Secretion of Tumor Necrosis Factor-Alpha. Crit. Care Med..

[176] Emani S., Ramlawi B., Sodha N.R., Li J., Bianchi C., Sellke F.W. (2009). Increased Vascular Permeability after Cardiopulmonary Bypass in Patients with Diabetes Is Associated with Increased Expression of Vascular Endothelial Growth Factor and Hepatocyte Growth Factor. J. Thorac. Cardiovasc. Surg..

[177] Serraf A., Aznag H., Baudet B., Détruit H., Séccatore F., Mazmanian M.G., Planché C. (2003). Pulmonary Vascular Endothelial Growth Factor and Nitric Oxide Interaction during Total Cardiopulmonary Bypass in Neonatal Pigs. J. Thorac. Cardiovasc. Surg..

[178] Gavard J., Gutkind J.S. (2006). VEGF Controls Endothelial-Cell Permeability by Promoting the β-Arrestin-Dependent Endocytosis of VE-Cadherin. Nat. Cell Biol..

[179] Lambeng N., Wallez Y., Rampon C., Cand F., Christé G., Gulino-Debrac D., Vilgrain I., Huber P. (2005). Vascular Endothelial-Cadherin Tyrosine Phosphorylation in Angiogenic and Quiescent Adult Tissues. Circ. Res..

[180] Feng J., Liu Y., Sabe A.A., Sadek A.A., Singh A.K., Sodha N.R., Sellke F.W. (2016). Differential Impairment of Adherens-Junction Expression/Phosphorylation after Cardioplegia in Diabetic versus Non-Diabetic Patients. Eur. J. Cardio-Thorac. Surg..

[181] Zhang Q., Feng R., Chaudhary O., Mahmood E., Baribeau Y., Rashid R., Khabbaz K.R., Chu L.M., Liu D.C., Senthilnathan V. (2021). Cardiopulmonary Bypass Suppresses Forkhead Box O3 and Downstream Autophagy in the Diabetic Human Heart. Ann. Thorac. Surg..

[182] Yu W., Gao B., Li N., Wang J., Qiu C., Zhang G., Liu M., Zhang R., Li C., Ji G. (2017). Sirt3 Deficiency Exacerbates Diabetic Cardiac Dysfunction: Role of Foxo3A-Parkin-Mediated Mitophagy. Biochim. Biophys. Acta (BBA)-Mol. Basis Dis..

[183] Xin Z., Ma Z., Jiang S., Wang D., Fan C., Di S., Hu W., Li T., She J., Yang Y. (2017). FOXOs in the Impaired Heart: New Therapeutic Targets for Cardiac Diseases. Biochim. Biophys. Acta (BBA)-Mol. Basis Dis..

[184] Sun D.-M., Yuan X., Wei H., Zhu S.-J., Zhang P., Zhang S.-J., Fan H.-G., Li Y., Zheng Z., Liu X.-C. (2014). Impaired Myocardium Energetics Associated with the Risk for New-Onset Atrial Fibrillation after Isolated Coronary Artery Bypass Graft Surgery. Coron. Artery Dis..

[185] Garcia L., Verdejo H.E., Kuzmicic J., Zalaquett R., Gonzalez S., Lavandero S., Corbalan R. (2012). Impaired Cardiac Autophagy in Patients Developing Postoperative Atrial Fibrillation. J. Thorac. Cardiovasc. Surg..

[186] Feng J., Liu Y., Dobrilovic N., Singh A.K., Sabe A.A., Guan Y., Bianchi C., Sellke F.W. (2013). Altered Expression and Activation of Mitogen-Activated Protein Kinases in Diabetic Heart during Cardioplegic Arrest and Cardiopulmonary Bypass. Surgery.

[187] Feng J., Liu Y., Chu L.M., Singh A.K., Dobrilovic N., Fingleton J.G., Clements R.T., Bianchi C., Sellke F.W. (2012). Changes in Microvascular Reactivity after Cardiopulmonary Bypass in Patients with Poorly Controlled versus Controlled Diabetes. Circulation.

[188] Kizub I.V., Klymenko K.I., Soloviev A.I. (2014). Protein Kinase C in Enhanced Vascular Tone in Diabetes Mellitus. Int. J. Cardiol..

[189] Feng J., Anderson K., Liu Y., Singh A.K., Ehsan A., Sellke F.W. (2017). Cyclooxygenase 2 Contributes to Bradykinin-Induced Microvascular Responses in Peripheral Arterioles after Cardiopulmonary Bypass. J. Surg. Res..

[190] Schoonen A., van Klei W.A., van Wolfswinkel L., van Loon K. (2022). Definitions of Low Cardiac Output Syndrome after Cardiac Surgery and Their Effect on the Incidence of Intraoperative LCOS: A Literature Review and Cohort Study. Front. Cardiovasc. Med..

[191] Chen A.L., Kindzelski B.A., Robinson J.P., Altshuler J.M., Schwann T.A., Vivacqua A. (2024). Deciphering Low Cardiac Output Syndrome: Insights and Management in Post-Cardiac Surgery. HSF.

[192] Álvarez J. (2008). Levosimendan and Low Cardiac Output Syndrome. Does Mortality Really Decrease?. Rev. Española Cardiol. (Engl. Ed.).

[193] Bielawska M., Warszyńska M., Stefańska M., Błyszczuk P. (2023). Autophagy in Heart Failure: Insights into Mechanisms and Therapeutic Implications. J. Cardiovasc. Dev. Dis..

[194] Khan T.A., Voisine P., Sellke F.W., Johnstone M.T., Veves A. (2005). Cardiac Surgery and Diabetes Mellitus. Diabetes and Cardiovascular Disease.

[195] Awad A.K., Elbahloul M.A., Al-omoush O., Abdelnasser O., Hajali M., Abdelnasser A., Saleh O., Altiti A., Elgharably H., El Diasty M. (2025). Impact of Postoperative Atrial Fibrillation (POAF) on Outcomes after Coronary Artery Bypass Grafting: A Meta-Analysis of Unique 247,270 Patients from 50 Studies. Am. Heart J. Plus Cardiol. Res. Pract..

[196] Duncan A.E., Kartashov A., Robinson S.B., Randall D., Zhang K., Luber J., James R.A., Halvorson S., Bokesch P. (2022). Risk Factors, Resource Use, and Cost of Postoperative Low Cardiac Output Syndrome. J. Thorac. Cardiovasc. Surg..

[197] Lomivorotov V.V., Efremov S.M., Kirov M.Y., Fominskiy E.V., Karaskov A.M. (2017). Low-Cardiac-Output Syndrome After Cardiac Surgery. J. Cardiothorac. Vasc. Anesth..

[198] Antunes P.E., Bernardo J.E., Eugénio L., de Oliveira J.F., Antunes M.J. (1997). Mediastinitis after Aorto-Coronary Bypass Surgery. Eur. J. Cardiothorac. Surg..

[199] Matata B.M., Galiñanes M. (2000). Cardiopulmonary Bypass Exacerbates Oxidative Stress but Does Not Increase Proinflammatory Cytokine Release in Patients with Diabetes Compared with Patients without Diabetes: Regulatory Effects of Exogenous Nitric Oxide. J. Thorac. Cardiovasc. Surg..

[200] Groom R.C., Rassias A.J., Cormack J.E., DeFoe G.R., DioDato C., Krumholz C.K., Forest R.J., Pieroni J.W., O’Connor B., Warren C.S. (2004). Highest Core Temperature during Cardiopulmonary Bypass and Rate of Mediastinitis. Perfusion.

[201] Voisine P., Ruel M., Khan T.A., Bianchi C., Xu S.-H., Kohane I., Libermann T.A., Otu H., Saltiel A.R., Sellke F.W. (2004). Differences in Gene Expression Profiles of Diabetic and Nondiabetic Patients Undergoing Cardiopulmonary Bypass and Cardioplegic Arrest. Circulation.

[202] Zakrzewski D., Janas J., Heretyk H., Stepińska J. (2010). Inflammatory Response and Postoperative Kidney Failure in Patients with Diabetes Type 2 or Impaired Glucose Tolerance Undergoing Heart Valve Surgery. Kardiol. Pol..

[203] Le Guillou V., Tamion F., Jouet I., Richard V., Mulder P., Bessou J.P., Doguet F. (2012). Mesenteric Endothelial Dysfunction in a Cardiopulmonary Bypass Rat Model: The Effect of Diabetes. Diabetes Vasc. Dis. Res..

[204] Doenst T., Wijeysundera D., Karkouti K., Zechner C., Maganti M., Rao V., Borger M.A. (2005). Hyperglycemia during Cardiopulmonary Bypass Is an Independent Risk Factor for Mortality in Patients Undergoing Cardiac Surgery. J. Thorac. Cardiovasc. Surg..

[205] Marty J.C., Bendhadra S., Amoureux S., Guilland J.-C., Vergely C., Rochette L., Girard C. (2008). Oxidative stress is exacerbated in diabetic patients during cardiopulmonary bypass. Ann. Cardiol. Angeiol..

[206] Mahmood E., Jeganathan J., Feng R., Saraf M., Khabbaz K., Mahmood F., Venkatachalam S., Liu D., Chu L., Parikh S.M. (2019). Decreased PGC-1α Post-Cardiopulmonary Bypass Leads to Impaired Oxidative Stress in Diabetic Patients. Ann. Thorac. Surg..

[207] Snel L.I.P., Li X., Weber N.C., Zuurbier C.J., Preckel B., van Raalte D.H., Hermanides J., Hulst A.H. (2024). Ketonaemia during Cardiopulmonary Bypass Surgery: A Prospective Observational Study. Br. J. Anaesth..

